# Wind energy conversion technologies and engineering approaches to enhancing wind power generation: A review

**DOI:** 10.1016/j.heliyon.2022.e11263

**Published:** 2022-10-26

**Authors:** Belachew Desalegn, Desta Gebeyehu, Bimrew Tamirat

**Affiliations:** a Energy Center, Faculty of Electrical and Computer Engineering, Bahir Dar Institute of Technology, Bahir Dar University, P. O. Box 26, Bahir Dar, Ethiopia; b Department of Physics, College of Natural and Computational Science, Wolaita Sodo University, P. O. Box 138, Wolaita Sodo, Ethiopia; c Department of Mechanical Engineering, Faculty of Engineering, Addis Ababa University, Addis Ababa, Ethiopia

**Keywords:** WECS technologies, PECs, ESS devices, Automated control strategies, Hybrid control algorithm, MBPC algorithm, MBD approach

## Abstract

Nowadays, engineers are toiling away to achieve the maximum possible wind energy harvesting with low costs through enhancing the performances of WECSs in efforts to realize the wind power future forecasts. In fact, achieving this is basically not an easy task due to the intricacies that partly stem from the stochastic nature of wind energy. Further, the efforts in this regard can also be impacted by the ongoing trends in various wind energy conversion-related technologies, and engineering approaches. Hence, the wind power optimization is determined depending on the types of WECS technologies, output power smoothing, and design development approaches that be employed. Currently, the variable speed operations-based WECS technologies are generally opted in wind farm applications. Meanwhile, power management system is the heart of a WECS, where smoothing output power with reducing costs could be implemented. On the other hand, the automated control strategies were reported in literatures to better optimize WECSs’ performances particularly in terms of costs compared to ESS devices. On this basis, MBPC and hybrid control algorithms were commonly presented as the current state-of-the-art for systems modeling, whereas MBD was preferred to be an efficient and cost-saving approach for advanced development of automated control systems. This study aims to conduct comparative analyses on WECS technologies (with different generators, and PECs) based on their energy harvesting capability, cost-effectiveness, and advances in designs. Assessments of the approaches and strategies for smoothing power production are also presented. Finally, the study concludes that trends in PECs, automated control strategies and MBD are the most compelling.

## 1 Introduction

Wind resource is ubiquitous, and it has been rapidly emerging as the efficient source of nonpolluting and inexhaustible energy for generating electric power across the globe. Indeed, electric power generation from wind resources has been undergoing varying levels of incremental improvements over the course of the last several decades in different regions of the world [1]. Nowadays, wind energy is second only to hydro (water) energy as the most powerful tributary of renewable and sustainable power in contributing to global electrification [2]. More importantly, wind power generation has also been predicted to sustain the remarkable growths in the future, in accordance with the emission goals that were set by UNCCC [3, 4]. Perhaps, different wind energy conversion technologies were developed and contributed for the achievement of the past and recent milestones in wind power generation. These technologies can be classified into different types based on some criteria, and their performances differ accordingly. For instance, based on their alignment to the ground [5], WECSs generally depend on either HAWTs or VAWTs, where HAWTs are extensively opted in wind power industry for their better wind energy harvesting performance. Moreover, depending on wind generator operating speed with reference to the fluctuating wind speeds [6], WECS technologies are usually classified as the constant-speed and variable-speed technologies. Based on this classification criterion, various types and topologies of wind generator technologies have been introduced for generating electricity from wind resources. The constant-speed-based SCIG; and variable-speed-based generator technologies such as DFIG, PMSG, and EES are among the most prominent in the modern wind farm industry.

The most recent WECSs generally depend on variable-speed generator technologies because of their outstanding efficiencies, and wider possibility for future enhancement. In the recent days, DFIG- and PMSG-based variable-speed WECS technologies are closely competing in the global wind energy commercial market [7]. Furthermore, the performance of WECSs also relies on the type of mechanical linkage between wind turbine and generator shaft: gearbox, and direct-drive technologies. For instance, among the leading variable-speed technologies, multiple- and single-gearbox systems with DFIGs are usually characterized to have low dynamic performance and high energy harvesting efficiency per cost whereas single-gearbox and direct-drive systems with PMSGs have high dynamic capability and superior power efficiency but the PMSGs-based WECSs are generally costly [8, 9, 10]. Yet, even though DFIG WECS has been recently reported to have better cumulative advantages, future trends of research studies indicated that PMSG WECS could become the leading choice for wind farm application as its operation is smoothly compatible with the extended voltage and power scales of its electrical conversion components [11, 12]. Hence, the optimization of the electrical components of PMSG-based WECS is one of the major themes of the recent and future research studies in the field of wind power engineering [13]. In the same time, EESG-based WECS is currently under continuous research studies for the better enhancement of its design efficiency in terms of cost, size, and weight though it is relatively less popular due to its cumulatively compromised performance in wind energy harvesting [9, 14].

Moreover, PECs have huge impact on the overall performance of the grid-connected WECS technologies. Among these technologies, the two-level (2L) – current source converter (CSC) [15, 16], and voltage source converter (VSC) [11, 17] topologies in back-to-back (BTB) configurations were conventionally being employed in small- and medium-scale wind farms for the last several decades; and they were usually compatible with DFIG-based WECS technology. Here, one of the main drawbacks of DFIG-based WECS is that it does not maintain operational compatibility with power converters of increasing power and voltage capacities [18]. Nevertheless, besides its considerable cost advantages, this technology is largely suitable for the vast application in small- and medium-scale onshore wind generation particularly with BTB 2L-VSC [19, 20]. On the other hand, modular multi-cell converter (MMC) [21, 22, 23] has been under continuous physical design development with different voltage capacities, and is recently being considered as the state-of-the-art particularly for application in PMSG-based wind farm industry with large-scale electricity production. The main attractive feature of PMSG-based WECS design is that the capacity of its power electronics converter can be scalable to increasing voltage levels, which makes the application of this technology highly desirable for multi-mega scale offshore wind energy deployment though its higher cost is still the major impediment. Several additional designs of converter technologies were also proposed in the multiple studies for applications in the wind power industries. These include DCC [20, 24], NPC [25, 26], ANPC [27, 28], etc., and they were introduced to be employed in the wind farms of large-scale power capacities that are mainly based on PMSG systems. In addition, similar studies were indicating that these converters are yet to be sufficiently matured for the smooth practical applications in the recent wind farm trends. Hence, significant improvements were suggested to be achieved subsequently in several aspects of the named converters’ limitations that are associated with operation and maintenance costs, weight, size, and power conversion capability.

On the other hand, enhancing wind power generation with WECS technologies has relied for many years now on the common trend of maximizing electricity generation whereby continual installations of wind power grid infrastructures are mandatory. For instance, this trend involves deploying of many wind farms across vast areas with the intention to capture wind resources over broader geographic ranges. Furthermore, various methods of design engineering have been implemented to enhance WECS technologies. Accordingly, increasing the radius of the swept area of wind turbine blades for extracting energy from a larger volume of air was one of the methods revealed to enhance WECSs design at component level [29]. However, increasing electric power generation should be realized with meeting important requirements in the processes of developing, and installing wind energy conversion technologies. This deems that power maximization demands should be considered in association with the amount of costs and time needed to develop and use WECS technologies in addition to the efficiency and reliability of the approach employed [30]. In this sense, enlarging wind turbine blades and reinstalling grid infrastructures are related to the physical prototyping-based engineering approach of enhancing wind energy harvesting technologies for harnessing maximum wind power from wind resources. But, this approach is not feasible for enhancing wind energy harvesting due to the fact that developing wind turbine physical systems in general, and enlarging the radius of the swept area of turbine blades in particular require much time, and high material costs. Besides, installation of wind farm infrastructure require large land resources, which bring another challenge to the process of power generation. Moreover, the physical prototyping-based design approach of developing WECSs has several serious drawbacks. In its stage of design development, it was reported that this approach [31] relies on the textual specification, which is ambiguous to analyze, and testing and validation processes could lead to erroneous results that not be reversible. It also pose complications to designing, and implementing the robust power management systems for WECSs.

Nowadays, the fundamental goal of enhancing WECSs is to broadening the scales of wind energy extraction from varying wind speed ranges for significantly maximizing electricity generation with remarkably decreasing costs. This principle of enhancing wind energy conversion should be met by ensuring the safety and integration of WECS technologies such as generators, power electronics converters, and grids. According to research reports [32, 33], WECS technologies have promisingly improved recently, and this has enabled to maximize wind power generation at fewer costs. In addition, researchers and engineers are still working to further improve the efficiency of WECSs in order to get further optimized output power with lower costs. Yet, power efficiency enhancement is obviously a demanding research problem as there are already several factors that contribute to influencing wind energy captures, which include wind generators, PECs, control systems, environmental conditions, etc. In the enhanced theories and practical operations, the electricity management systems [34, 35] were proven to be the heart of the variable-speed WECS technologies; and therefore, the implementation of efficient management systems for electric generators and PECs can have a significant impact in increasing wind energy harvesting, and hence enhancing WECS efficiencies. On account of the intermittence characteristic of wind speed, the wind power generation during the operation of WECSs (generators, PECs, etc.) is fluctuating constantly, dramatically and rapidly. Due to this, a number of profound challenges were reported to be resulted from the power systems disturbances, which include [36, 37, 38, 39]: the grid frequency variation, the real power disturbance, and the voltage flicker at the buses of the power grid. In other words, this creates the degraded output power quality and instability in the WECSs.

In general, wind energy has a considerable influence on the dynamic behavior of power systems during regular operations and abnormal conditions with increasing penetration into the grid system. Particularly, the study of the influence of wind power on WECS transient stability has become a crucial research issue nowadays [40]. This implies the main challenge in the operation of the WECSs is the robust performance under transient fault conditions. On one hand, different power smoothing options were reported to tackle the outlined problems such that a number of options would apply the ESS devices that include batteries, flywheel, compressed air storage, and so forth [41, 42]. However, implementing ESS devices is generally not a desirable option due to the fact that these devices add high extra costs to WECSs even though they were proven to show good performance in smoothing output power. Consequently, the economical and robust power smoothing system should be developed in place of the applications of ESS devices. Accordingly, a virtual system was widely favored by multiple engineering studies [43, 44, 45, 46], and it can be built through implementation of various automated control strategies, which would ultimately regulate different operating parameters of WECSs. Recently, a dual objectives‒control technique was presented to reduce the torque ripples of the turbine shaft by implementing the frequency separation principle [47, 48]. In addition, the real current control method [49], the generator torque control strategy [50], real and reactive power control [51], and independent pitch control [52] were employed to streamline the generator output power. Moreover, the kinetic energy optimization-based inertial control strategies [46, 53] were demonstrated through simulation to be identified as outstandingly outperforming virtual power smoothing approach.

Ultimately, reliable, efficient and effective control strategies are required to be designed in WECSs to maintain systems’ comprehensive performance. In this sense, different control design strategies can be implemented to enhance WECS technologies for the reduced costs and smoothed output power. More importantly, hybrid [54], and model-based predictive [55] control design strategies were highly recommended in recent studies due to their robust performances that would enable them to circumvent the nonlinear and unpredictable characteristics of WECSs operations. Hybrid control strategies were demonstrated in [56] as being designed by combining hard control that includes proportional integral derivative (PID), sliding mode control (SMC), adaptive control, etc., and soft control that involves fuzzy logic control (FLC), neural network control (NNC), genetic algorithm (GA), etc. so as to make use of the cumulative advantages of hard and soft control strategies by reducing the control complexity of the systems in improving efficiency and dynamic stability. In practical applications, hybrid design strategies could optimize the systems by alleviating the respective limitations of PID, SMC, FLC, NNC, etc. and by fusing their respective advantages. Furthermore, the fusions could also possibly be made between soft and soft controls, whereas the hard and soft combinations were characterized in some studies [57, 58] as more efficient strategies. Similarly, model-based predictive control (MBPC) was prevalently demonstrated by the recent research works [59, 60, 61] as the advanced strategy having appealing features, which can be utilized to develop efficient and cost-effective power smoothing system. In general, the ultimate goal of implementing these strategies (including hybrid and MBPC) are to establish the stringent power control systems that eventually meet advanced operation requirements (power reliability, FRT capability, maximum power production, overall cost optimization) for WECS technologies. Moreover, these control design strategies can enhance WECS technologies by reducing their overall design complexities, and thereby achieving rapid dynamic and transient responses.

In the end, the WECSs control design strategies can be developed and evaluated by employing several different approaches. Basically, the model-based design (MBD) approach was introduced against that of physical prototyping to smooth the design development and optimization processes in the particular case of complex systems including WECSs. In a number of recent studies [62, 63, 64, 65], MBD was reported to be methodologically effective and efficient particularly for modeling and evaluating WECSs control system designs based on the proposed control design strategies. In the typical case, a WECS control system model can be simulated, tested, and preliminarily validated based on a model predictive algorithm and by using MATLAB/SIMULINK software platform, external target computer, and controller. On the other hand, a complete confirmation of a WECS design's real performance would be challenging according to advanced research reports [66, 67, 68]. Yet, regardless of its limitations, the MBD methodology was generally considered by large study projects [69, 70] as a compelling approach as it could ultimately enable to achieve the efficient and reliable wind energy conversions, which will result in more energy transferred to the electrical power systems without needing to build complex infrastructures, and for the similar scales of energy extracted from the wind resources.

The main objective of this study is conducting a comprehensive assessment on the most recent wind power generation-based – technology systems (turbine generators and PECs) and engineering approaches in a manner that it will have a potential contribution in helping to inspire further studies in the future. Accordingly, it aims to explore the operating characteristics of the WECSs, such as those that are based on: SCIG, DFIG, PMSG, and EESG by identifying the most advanced designs for wind farm applications. Here, the basic comparison metrics including energy harvesting efficiency, capital cost, power reliability, and FRT capability are being considered to demonstrate the recent trends in these systems. As the core technologies and component parts of the WECSs, this study also discusses the development trends of various PECs for enhancing the performances of the recent and future wind farms. Furthermore, as the keys to smoothing WECSs operations for the enhanced electricity generation, two power engineering approaches that are based on the ESS devices, and the automated control strategies/virtual systems are examined based on various standards including recent and future research perspectives. Conclusively, MBPC strategy is earned a special consideration; and as the emerging approach for developing and evaluating a design of a WECS's core component (control system), MBD methodology is demonstrated.

## 2 WECS technologies

**Figure 1:**
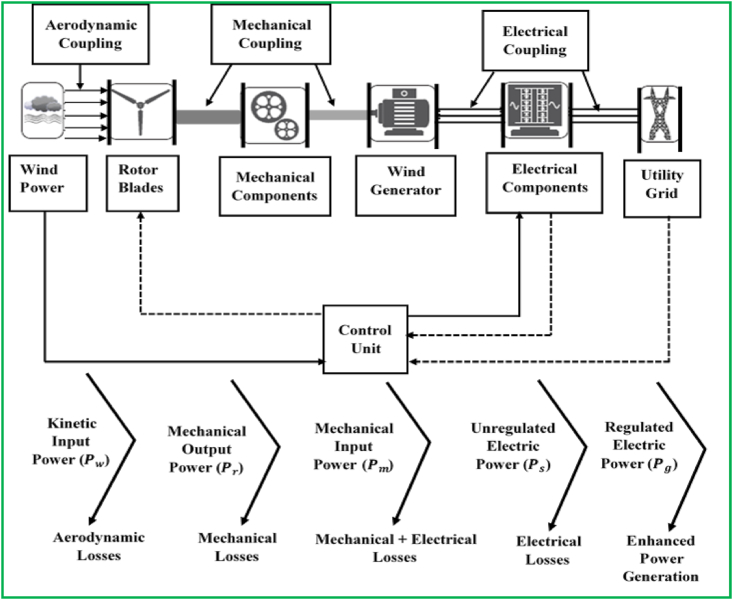
Typical wind energy conversion networks and power transformation phases for enhanced electricity generation.

Wind energy harvesting technologies [8, 71, 72] are configured to harness the energy of wind movement for generating electric power by employing various mechanical and electrical subsystems such as wind turbine rotors, generators, control systems, and the interconnection apparatuses such as possible PECs and transformers. The principal components of the present-day wind turbines are the tower, the rotor, and the nacelle, which accommodate the transmission mechanisms and the generator. The wind turbine harnesses the kinetic energy of wind in the rotor composed of two or more blades systematically tied to an electrical machine or generator. The main module of the mechanical design is the gearbox, which transfigures the inadequate spinning speeds of the wind turbine to considerable spinning speeds on the electrical machine side. The spinning of the electrical machine's shaft run by the wind turbine produces electric power, whose output is preserved according to specifications, by making use of desirable control and supervision strategies. In addition to managing the electrical outputs, these control units also involve protection schemes to protect the overall system from the possible damage that could be caused by the sudden electrical circuit faults. The general structure of WECS is illustrated in Figure 1. In general, the energy transforming network can be structured as four main units [73]: aerodynamic unit, comprising primarily the turbine rotor, which is made up of blades, and turbine hub that is the bearer for blades; drive train, usually consisting of: slow-speed shaft – tied to the turbine hub, speed enhancer and maximum-speed shaft – running the electrical generator; electromagnetic unit, comprising basically of the electric generator; and electric component, involving the devices for grid integration (power electronics converter, transformer, etc.), and local grid.

### 2.1 Classifications of WECSs

WECS technologies can be divided into various classifications on the basis of different criteria or factors. According to [6, 8, 10, 74], the most popular classification factors include: (i) WECS electric output power scale (small, moderate, and large power), (ii) aerodynamic power control strategy for strong wind-speed characteristics (stall pitch control), (iii) configuration of wind generator shaft with reference to the installation ground (HAWT and VAWT), (iv) type of system to deliver the electric output power (autonomous and grid-tied), (v) wind generator applicable speed with reference to the changing wind speeds, (vi) site for installation of WECS (onshore and offshore), (vii) type of mechanical integration across the turbine and generator shaft (with gearbox and direct-drive), and (viii) wind speed velocities (slow, medium, and maximum) impacting the WECS. The overall quality of wind energy conversion is generally not satisfactory with VAWTs, and hence, the modern commercial WECSs implement HAWTs with three rotor blades operation. Besides, depending on wind generator applicable speed with reference to the changing wind speeds, WECSs can be designed for either a constant (fixed) speed application (FSWT), or for the variable-speed operation (VSWT), which has cumulatively superior energy conversion performance. Further comparisons are made among HAWT vs. VAWT, and FSWT vs. VSWT in Table 1. For instance, variable-speed WECS has outstanding energy harvesting quality with minimized mechanical stress and lessened noise. Moreover, variable-speed WECSs generate higher power than fixed-speed one, in comparison, but it necessitates advanced power converters, control devices to offer constant frequency and fixed power factor, and this raises the system complexity. This paper primarily deals with variable-speed-based HAWT (WECS) technologies.

**Table 1:** Advantages (✓) and limitations (✗) of WECSs based on: the alignment of wind generator shaft with reference to the ground, and wind generator applicable speed with reference to the changing wind speeds.

WECS types	Advantages	Limitations
HAWT [75, 76, 77]	✓ Robust in converting wind energy to electrical output power ✓ Preferable for accessing to reliable wind energy extraction	✗ Increased probability of system failure and maintenance due design complexity ✗ The wind direction adjustment is mandatory
VAWT [78, 79, 80, 81]	✓ Suitable for installation and maintenance due to simplicity of its configuration ✓ Not reliant on the direction of wind for effective operation	✗ Insufficient capability of wind energy harvesting ✗ Responsible for increased torque ripples and susceptible to mechanical disturbances
FSWT [6, 82, 83]	✓ No complexity in structure, sually not prone to failures, reliable ✓ Reduced installation and maintenance costs	✗ Comparatively low energy harvesting capability ✗ Maximum fatigue loads ✗ Inferior power quality to the grid
VSWT [84, 85, 86]	✓ Superior wind energy harvesting efficiency ✓ Enhanced power quality and stability ✓ Minimized mechanical fatigue loads	✗ Extra cost due to making use of converters, which result in electrical losses ✗ Highly sophisticated control system, which adds complexity to design process

### 2.2 Configurations and features of prominent wind power generation systems

The wind electric power generation network comprises electromagnetic and electrical subsystems inseparably. In addition to the electrical generator and power electronics converter it usually includes an electrical transformer to establish the grid voltage compliance. Nevertheless, the design structure of power generation system relies on the type of WECS and on its grid interface. The WECSs can have various configurations. Accordingly, in [5, 87], the general configurations of WECSs were named based on a blending of two criteria: (1) the electric generator applicable speed with reference to the changing speed and (2) the type of mechanical integration across turbine and generator shaft. These configurations include:

A. Constant Speed WECS with triple-stage Gearbox.
B. Partial-scale variable speed WECS with triple-stage gearbox.
C. Full-scale variable speed gearless WECSs.
D. Partial-scale variable Speed WECS with a Single-stage Gearbox.
E. Full-scale variable Speed WECS with a Single-stage Gearbox.

Based on the stated criteria and under the general configurations listed above (from A to E), there are some specific WECSs that were considered as popular in [9]. The structures, advantages and drawbacks of these WECSs are briefly discussed under subsections to follow. The comparisons between advanced wind energy conversion technologies are also demonstrated based on the main requirements of electricity generation in Table 2.

**Table 2:** Qualitative comparisons of various variable-speed technologies: TG-DFIG, DD-EESG, DD-PMSG, SG-PMSG and SG-DFIG WECSs.

Metrics	TG-DFIG	DD-EESG	DD-PMSG	SG-PMSG	SG-DFIG
⁃ System cost [9, 106]	Relatively lower than the rests	Highest of all	Lower than DD-EESG but higher than the rests	Lower than DD-EESG and DD-PMSG and higher DFIGs	Relatively lower than TG-DFIG
⁃ Power yield [5, 9, 107]	Relatively lower than the rests	Slightly higher than TG-DFIG and SG-DFIG and nearly equal to SG-PMSG	Higher than all the rests	Slightly higher than TG-DFIG and SG-DFIG but lower than DD-PMSG	Relatively Higher than TG-DFIG
⁃ Power yield/cost [8, 19]	Slightly lower than SG-DFIG but higher than DD-EESG and DD-PMSG	Lower than the rests	Higher than DD-EESG and slightly lower than the rests	Higher than DD-EESG and DD-PMSG and slightly lower than SG-DFIG	Higher than the rests
⁃ Reliability [9, 87, 108]	Low	In the middle	Higher than the rests	Higher next to DD-PMSG	In the middle
⁃ FRT capability [19, 74]	Weak	Strong	Strong	Strong	Weak

#### 2.2.1 Squirrel cage induction generator with triple-stage gearbox (TG-SCIG)

**Figure 2:**
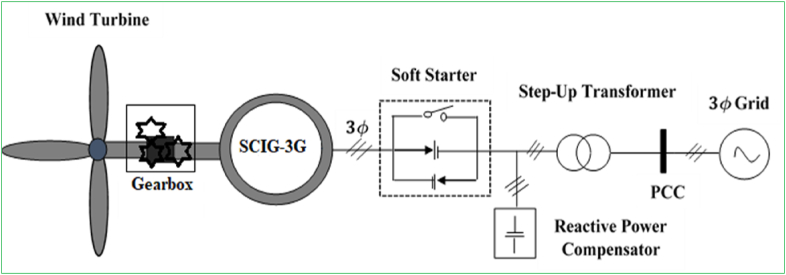
Structure of a constant-speed WECS with triple-stage gearbox SCIG.

The TG-SCIG system is depicted with exclusion of a power converter component in Figure 2, where the generator is tied to the grid via a soft starter and coupling transformer. This system is the traditional and it was employed in wind energy industry since the very beginning of starting to harness wind resources. The main benefits and limitations of this system design are [88, 89, 90]:

✓ Low complexity of energy harvesting structure.
✓ Reduced start-up and operation costs due to its cheap component, low-cost soft starter.
✓ Stable operation since power converter is not required.
✗ Insufficient wind energy harvesting capability due to limited (1%) speed scale.
✗ Intermittence characteristics of wind speed result in grid frequency fluctuations.
✗ Grid disturbances generate high stress on its mechanical subsystems.

#### 2.2.2 Doubly-fed induction generators with single-stage and three-stage gearbox (SG-DFIG and TG-DFIG)

**Figure 3:**
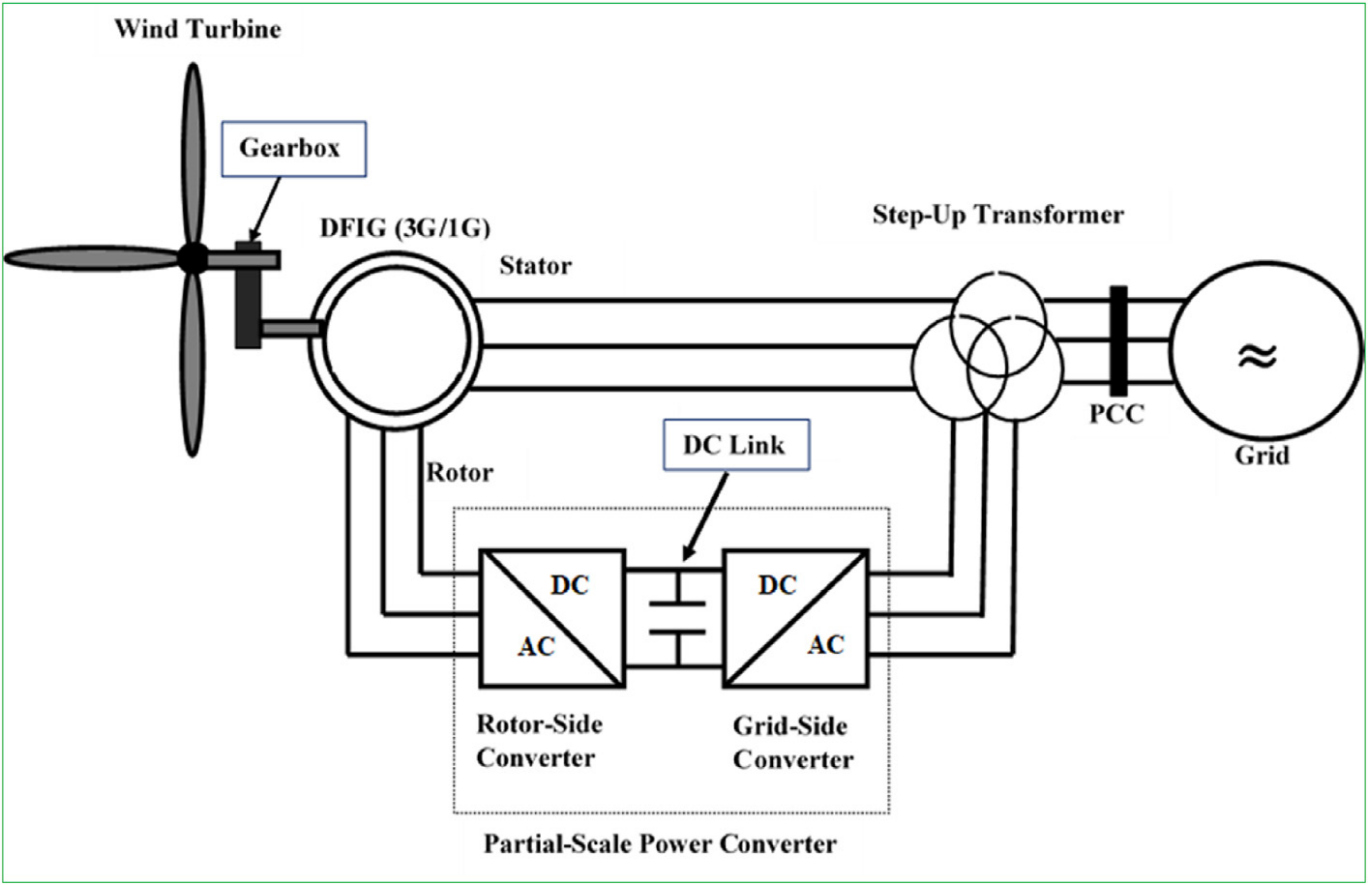
Configuration of wind energy harvesting system with DFIG and partial-scale power converter.

The general structure of WECS incorporating a DFIGs and power electronic converter is illustrated in Figure 3. As its naming hints, energy harvested by the DFIG is delivered to the grid via stator and rotor windings. The converter in the rotor circuit is designed to manage entirely the slip power; hence, the conversion efficiency of this system is limited to 30% of the electric generator real power. Employing a partial-scale (30%) converter has the benefits of minimizing cost, weight, and nacelle space necessity. The power converter generally comprises two-stage voltage source converters (VSCs) tied in a BTB configuration. A rotor-side converter (RSC), regulates the generator torque/speed or real/reactive power, while the GSC handles the net DC-bus voltage.

Furthermore, since the rated power of the converter for both DFIGs with single- and triple-stage gearbox is only 30% of the systems, this presents the special advantages in terms of start-up investment and energy harvesting performance as opposed to the technologies with the full-range power converters, particularly EESGs (Section 2.2.3). On the other hand, because of the only one-level of speed maximizing, the generator speed is appreciably low, whereas the torque is appreciably high, and therefore, the SG-DFIG needs to be designed with an increasing diameter and air gap. This sequentially results in the generation of substantial magnetizing current and considerable power losses. The main benefits and drawbacks of DFIG-based wind energy harvesting technologies are summed up below [7, 12, 91, 92]:

✓ The power converters enables two-way power transport in the rotor circuit. The generator speed can be synchronized 30% greater than or less than the synchronous speed. Hence, the energy harvesting capability is outstanding and fatigue loads on the mechanical subsystems is insignificant.
✓ The power converter works as a smoothing solution for grid integration and grid-side reactive power reserve. Hence, soft starters and capacitor banks are not required.
✓ The power converter additionally offers superior dynamic capability and reliability by alleviating power system instabilities in contrast with TG-SCIG.
✗ Increase in the system installation investment and its design complexity due to incorporation of the power electronics converter.
✗ Incompatible for offshore wind industries due to the consistent maintenance requirement by the slip rings and brushes in DFIG with the triple-stage gearbox.
✗ FRT tractability is challenging due to the straight grid-coupled DFIG stator terminals and partial (30%) load power converter.

#### 2.2.3 The direct-drive WECS with an electrically excited synchronous generator (DD-EESG)

**Figure 4:**
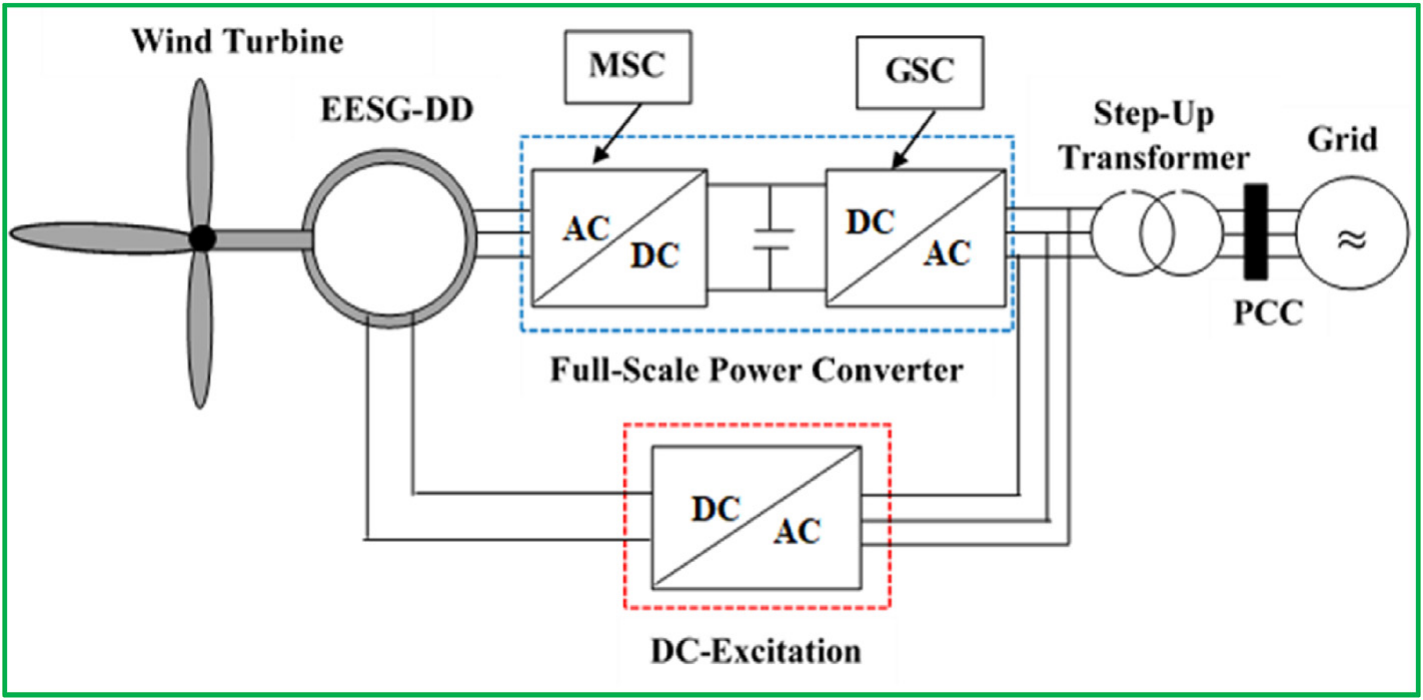
Structure of a gearless wind energy harvesting system with EESG and full-load converter.

The gearless variable-speed WECS with the EESG and full-load converter is illustrated in Figure 4. The design of DD-EESG is developed with a rotor accompanying the field system equipped with a DC activation. The generator should be configured with an increasing number of poles to enhance the ungeared system. As a result, the volume and weight of this slow-speed generator is highly larger compared to those of the triple-stage gearbox-generators, namely TG-SCIG and TG-DFIG. The slip rings and brushes are essential in the DD-EESG for activating windings which raise the necessity for system maintenance. Besides, the field winding result in power losses, and, thus deteriorating the system performance. Furthermore, the cons and pros of DD-EESG are briefly summarized as follows [9, 14, 93]:

✓ Full-scale PEC allows it to completely handle the frequency and amplitude of the voltage on the machine side.
✓ Relatively generates high electrical power compared DFIG WECSs.
✓ Produces reduced noise due to the reason that it is gearless.
✗ Considerable system cost at installation level because of the use of costly electronic components.
✗ Requires the application of a DC source with brushes and slip rings for the excitation of rotor winding.
✗ Bigger size of geometric shapes and massive generator weight.

#### 2.2.4 The single-stage gearbox and the direct-drive with permanent magnet synchronous generators (SG-PMSG and DD-PMSG)

**Figure 5:**
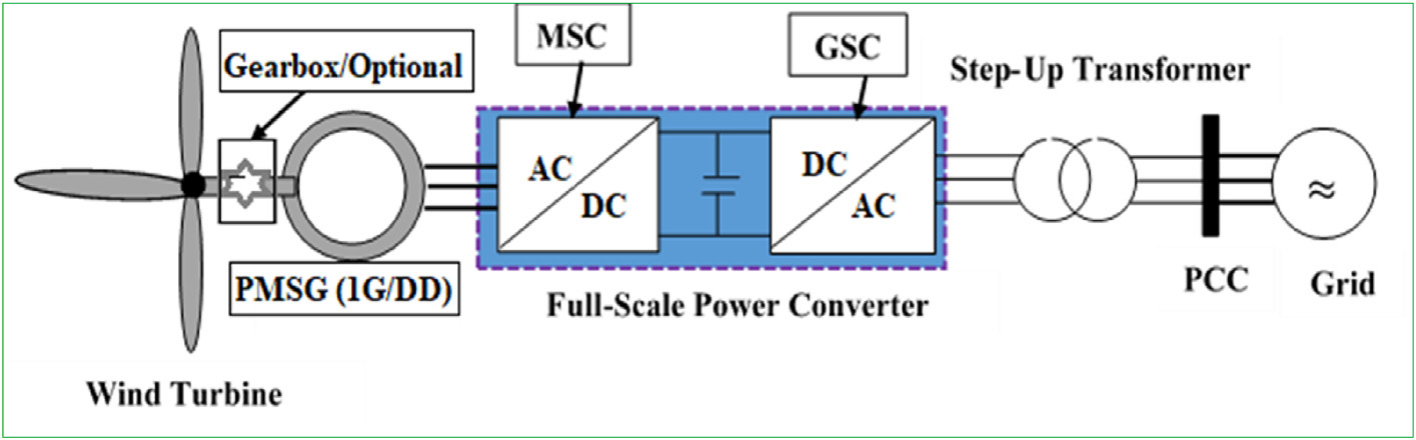
Configuration of wind energy harvesting system with PMSG and full-scale power converter.

The configuration of grid-connected PMSG (single-stage geared and gearless) with a wind generator and a full-range power converter comprised the electric machine-side converter (MSC), DC-link capacitor, and GSC is depicted in Figure 5. As opposed to DFIG-based WECSs for which the power converter is tied in the rotor circuit to generate slip power, PMSG-based WECSs use a power converter across the wind generator stator terminals and power grid to run all the electric power generated. Hence, the efficiency of the power converter is raised from 30% to 100%. The commercial price of a power converter in DFIGs and WECSs with full-scale variable-speed power converter including those based on PMSGs and EESGs is nearly 5% and 7–12% (based on the version of converter technology) of the entire each WECS price, respectively [8]. A full-load (100%) power converter results in a full-variable- speed scale (0%–100%) and the power generated by PMSG WECSs is exceedingly high. Compared to the groups of wind generators, PMSG is the most prominent in variable-speed WECSs with full-scale power converters.

Moreover, the gearless PMSG WECS is the most promising technology to date. Unlike direct-drive EESG technology, the external activation and slip rings are not required in gearless PMSG system, thus its energy harvesting capability and dynamic performance is better compared to EESG. Besides, in comparison with single-stage and triple-stage geared WECSs, advantage of DD-PMSG is that turbine noise is minimized since it is the gearless technology with independent activation system. However, until the recent moment, it was not feasible for the wind industry to design wind generators with increased external diameter due to the logistics and construction technology complications, which restrict the advancement of the ungeared WECSs with high MW power scale.

The step-up transformer can be avoided in PMSG WECSs by adjusting the power converters at a MV scale. In general, the main advantages and disadvantages of the full-scale power converter PMSG technologies are the following [8, 9, 59, 60, 94]:

✓ Outperforming energy harvesting efficiency and no fatigue load on mechanical subsystems due to implementation of full-load (0–100%) application.
✓ Autonomous real and reactive power regulation help to maintain outstanding FRT capability.
✓ The electric machine is entirely detached from the grid. The power converters additionally ensure smooth grid integration.
✗ Due to the full-scale power converter, the start-up cost and nacelle space necessity as well as the whole system sophistication rise.
✗ Increasing power losses in the converter deteriorate the whole power system performance.
✗ The sophistication of digital control system design for power converters escalates.

### 2.3 Variable-speed power generation systems (DFIGs, PMSGs and EESG WECSs): operational characteristics and research problems

According to the discussions that have been made so far (under Sections 2.2.1 to 2.2.4), the variable-speed WECSs, which are based on DFIGs, PMSGs and EESG generally seem to have better overall performance compared to traditional SCIG. On the other hand, the comparisons among triple-stage gearbox (TG)-DFIG, direct-drive (DD)-EESG, direct-derive (DD)-PMSG, single-stage gearbox (SG)-PMSG and single-stage gearbox (SG)-DFIG are briefly summarized in Table 2 by considering the fundamental operational characteristics of machines such as cost, power yield, power yield/cost, reliability, and FRT capability as metrics. It can generally be interpreted that the performance of DD-EESG is moderately good, whereas DFIGs and PMSGs are cumulatively high-performing according to the implemented metrics or criteria, and thus based on the main objectives of this study. In addition, the summaries of research problems aiming at studying the outlined limitations of these WECSs are presented in Table 3 in terms various layouts of power generation systems that are generally rely on DFIG, PMSG, and EESG technologies.

**Table 3:** Summaries of recent research problems aiming at improving the limitations of each WECS outlined in Table 2.

Proposed WECSs	General research problems	Description of specific problems	Ref.
Based on DFIG technology	Enhancing system's FRT capability and protection	Enhancing power quality of grid connected system through the employment of low voltage ride through (LVRT) scheme that was designed as capacitor-inductor series connection and capacitor/inductor-resistor parallel connection.	[95]
		Estimating the impact of rotor current attenuation process on the power generation stability and devices based on the real-time data service (RTDS) and physical controller of converter in helping to enhance power quality by maintaining the safety protection for power devices.	[96]
		Enhancing power quality by employing non-superconducting fault current limiter based on bridge-type flux coupling method.	[97]
		Ensure to maintain system's continuous operation during voltage dips (low voltage ride through enhancement) by employing external retrofit and internal control techniques.	[98]
		Compensating voltage swell by limiting the fault short circuit current through the application of dynamic voltage resistor (DVR)-FLC technique in ensuring to develop robust system of enhanced power quality.	[99]
	Improving power reliability	Reducing the model complexity of different DFIG-turbine systems by making use of the novel model reduction margin (MRM) as optimization strategy and the New England test system (NETS) as evaluation model; while evaluating the damping torque contribution to stability margin from the dynamic model components of these systems.	[100]
		Employing an enhanced primary frequency response (PFR) strategy so as to reduce the pitch angle to slowly feed the active power to the grid system in improving the frequency stability of the system.	[101]
		Enhancing the capability of frequency regulation by considering the interdependences among the variables including rotor speed, rotor current frequency, and power system frequency by using a novel control strategy applied to maximum energy harvesting.	[109]
Based on PMSG technology	Optimizing cost and reliability	Implementing swap control scheme that facilitates to use the turbine-generator rotor inertia for storing surplus power during grid voltage dips, which ultimately helps to achieve the removal of extra hardware devices; and ensure operation compatibility, lowering size, cost and switching losses of the system.	[102]
		Reducing chattering problem, enhancing system's operation reliability, increasing its lifespan, and thus optimizing its cost by regulating the generator and grid-side converter through implementation of an enhanced power smoothing strategy based on continuous switching control.	[103]
		Solving the intricacies associated with the transient power stability by considering the insulated gate bipolar transistors' (IGBTs') excitation parameters, and employing a severe three-line-to-ground fault scheme.	[104]
		Realizing a fast transient response to smooth operation of the system by employing different optimization strategies with the evaluation model based on braking chopper (BC).	[105]
Based on EESG technology	Optimizing design	Developing the robust control system design under the consideration of the wind turbine mechanical resonance, and by implementation the resonant damping control strategy for rotor speed and torque.	[14]
		Enhancing low voltage ride through (LVRT) based on the provision of: active power in proportion to the voltage retained during voltage dip, and maximum reactive current until the voltage starts recovering.	[93]

Multiple recent research studies were largely focused on introducing different methods that could be pursued to enhance the power performances of particularly the DFIG- and PMSG-based WECSs, as it can also be observed from the outlines presented in Table 3. Accordingly, DFIG-based WECS with varying power capacities was proposed to be enhanced by improving its FRT capability along with maintaining its operation safety based on different methods and protection schemes. For instance, the power quality of the grid connected DFIG system was considered to be enhanced by employing low voltage ride through (LVRT) strategy that was developed as capacitor-inductor series connection and capacitor/inductor-resistor parallel connection [95]; the impact of rotor current attenuation process on the DFIG system components and power stability was studied based on the real-time data service (RTDS) and physical controller in enabling the enhancement of power quality and power devices protection [96]. In addition, non-superconducting fault current limiter that was based on bridge-type flux coupling method [97]; external retrofit and internal control techniques [98]; and dynamic voltage resistor (DVR)-FLC technique [99] were also proposed to enhance FRT capability based on the modeling of DFIG system components. Moreover, the power reliability with DFIG system operations was reported to be improved based on the implementation of different research modeling strategies including: a novel model reduction margin (MRM) & New England test system (NETS) [100]; an enhanced primary frequency response (PFR) [101]; etc. as it can be seen from Table 3.

Various modeling strategies were also proposed by different studies to enhance power reliability and optimize the overall cost for PMSG-based WECS as indicated in Table 3. For instance, swap control strategy was proposed to be implemented to facilitate the application of turbine generator rotor inertia for storing maximum power during the occurrence of grid voltage dips [102]. The most interesting part of this strategy is that it helps to realize the elimination of extra hardware devices by ensuring system's operation compatibility along with reducing its size, cost, and switching losses. Further, chattering problems associated with PMSG system was reported to be minimized so as to enhance power reliability, increase system's lifespan, and thus optimize its cost by implementing continuous switching control strategy [103]. A severe three-line-to-ground fault [104]; and braking chopper (BC) [105] schemes were also implemented to smooth the operations of PMSG-based WECS. On the other hand, only few studies were recently introduced based on the design optimization of EESG wind power generation system, and two of them are similarly presented based on [14], and [93].

### 2.4 Power electronics converters advances for wind farm applications

Power electronics converters (PECs) have become the crucial components of the WECSs particularly that which rely on the variable-speed and grid operations. In general, PECs play a prime role in the wind farm applications such that their overall performances can be further enhanced in helping to achieve the most important and immediate objectives of wind power production. Accordingly, the objectives of developing PECs should be ultimately aiming at minimizing the costs of wind power, ensuring the energy harvesting on the broader wind speed ranges, improving the power reliability, creating the fault-resilient WECSs, reducing the weight and footprint of the WECSs, attaining excellent output power quality, and enhancing the grid integration with the stringent grid codes. The uses of power converter technologies in WECSs are stated as below:

⁃ A soft starter is used in TG-SCIG WECS for smoothing grid integration by alleviating startup in-rush currents [6].
⁃ A partial-scale converter is used in DFIG-based WECSs for managing slip power and raising speed range in the application [110].
⁃ The full-scale converters are used in PMSG-based WECSs for decoupling the generators from the grid and offering a full-speed scale operations [111].
⁃ A full-scale BTB converter is applicable to regulate the DC-excitation in EESG WECS so that the output voltage and frequency of machine comply with the grid characteristics [112].

In addition, PEC technologies have gone through significant improvements, and the most advanced technologies are based on the full-scale large-power operations. Nowadays, the wind industries widely implement the applications of PEC technologies in power generation systems and wind farms for achieving the maximum possible wind energy harvesting along with enhanced grid integration. The function of PECs is to advance variable-speed applications prominently in DFIG and PMSG WECSs while avoiding the necessity of a soft starter and reactive power balance. To ensure the grid integration of the stated WECSs, the unsteady voltage/frequency of the wind generator must be transformed into a steady voltage/frequency. Hence, a broad option of energy transforming levels can be implemented by different converter topologies. Larger number of these energy transforming levels have earned commercial applications, whereas others have been recommended in studies with interesting features for future advancement, while still the rests have been introduced from the variable-speed electric drives industry. Power converters are mainly grouped as direct and indirect: direct conversion employs single-stage AC/AC power converters, while indirect conversion employs two-level (AC/DC + DC/AC) or three-level (AC/DC + DC/DC + DC/AC) power converters.

**Figure 6:**
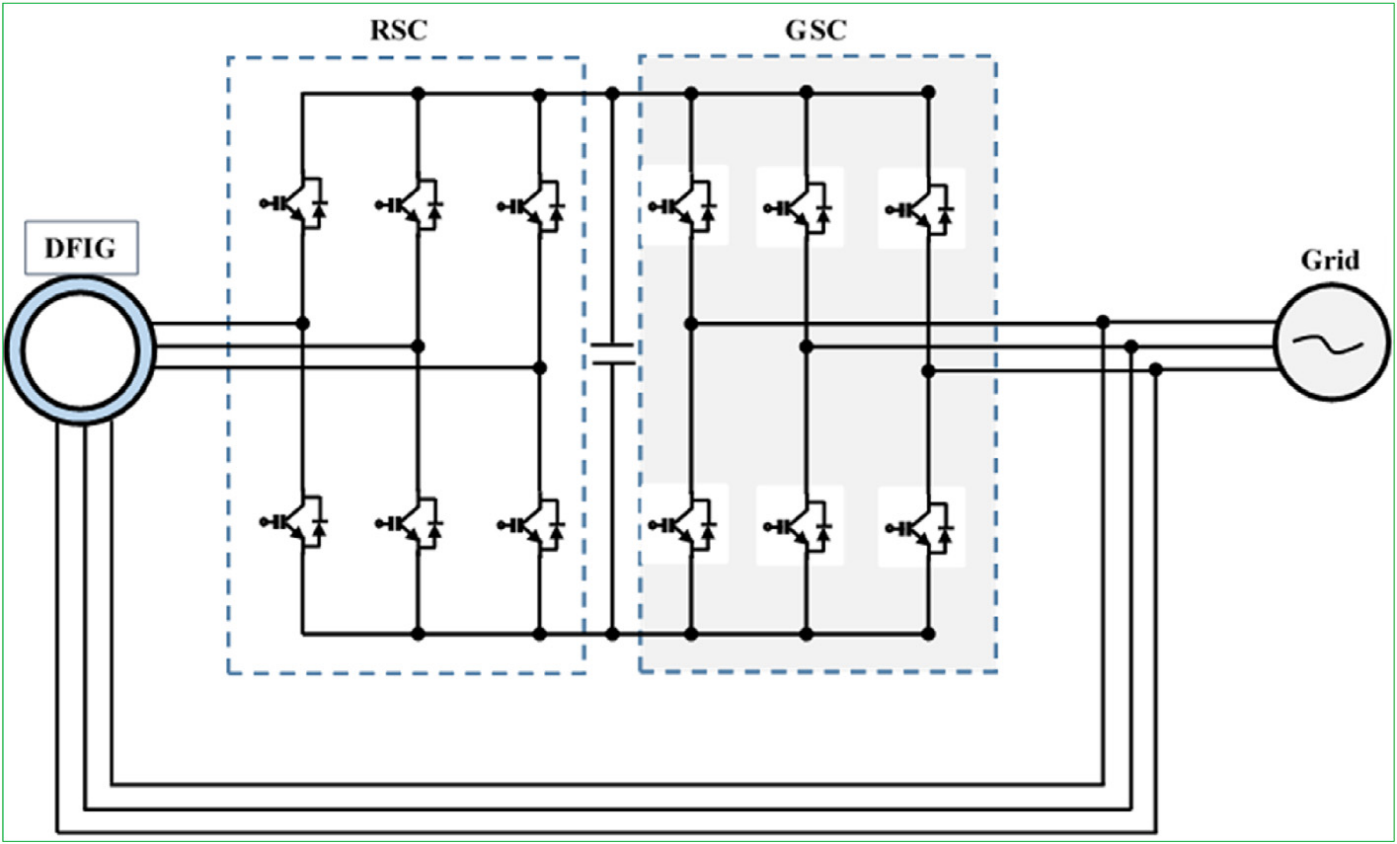
2-L BTB VSC configuration for DFIG-based WECS.

**Figure 7:**
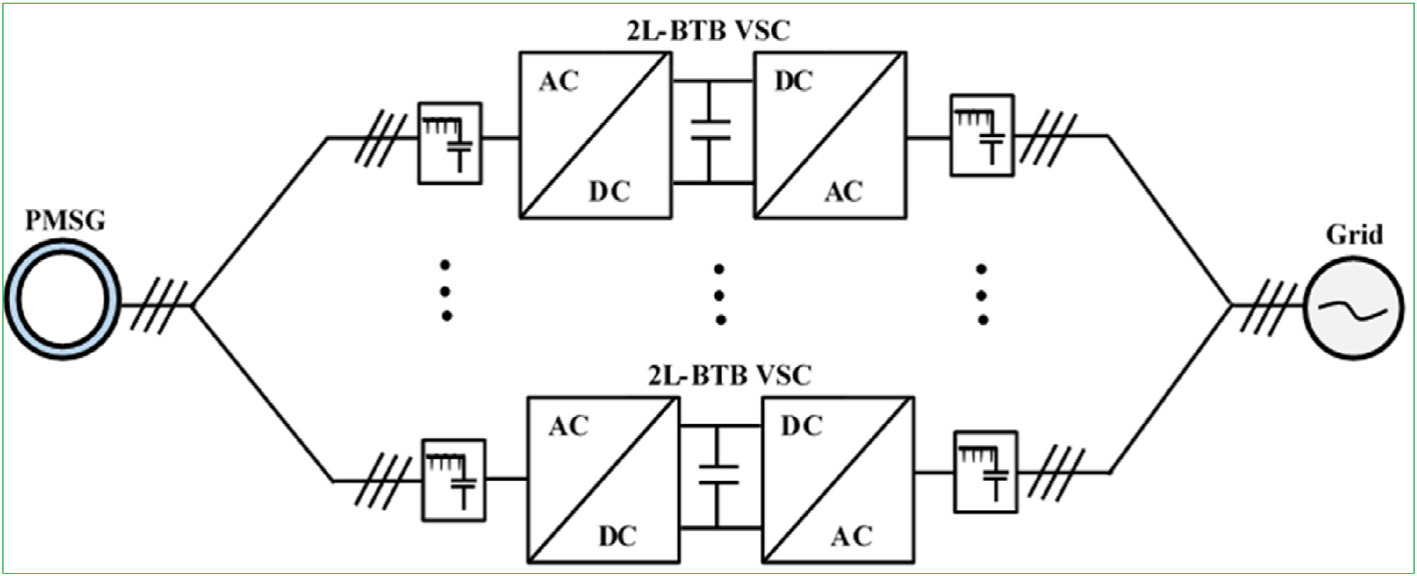
Multi-cell 2L-BTB VSC for PMSG-based WECS.

Direct AC/AC (matrix) power converters battle with two-level (AC/DC + DC/AC) VSCs in the electric drives industry due to the removal of DC-link devices and exceeding robustness. Indirect two-level (AC/DC + DC/AC) power converters are largely incorporated in a back-to-back (BTB) tied configuration. An entirely regulated AC/DC power converter, DC-link devices that include capacitors and inductors, and a DC/AC converter make up the BTB configuration. BTB converters can be implemented as either VSCs or CSCs. For the wind energy generation application, BTB VSCs are efficient, economically advantageous, and robust. Another important feature of BTB VSCs topology is that they are compatible for both DFIG and PMSG WECSs at small and medium-power scales. The conventional two-level (2L) BTB VSC configuration is compatible for DFIG-based small/medium-power scale WECS (Figure 6), whereas the multi-cell 2L-BTB VSC parallel configuration is the state-of-the-art technology for PMSG-based medium-power scale WECS (Figure 7). These converters smooth (characteristically) a four-quadrant application with a comparatively uncomplicated design layout.

For multi MW-power scale wind farm industries, high-voltage converters are preferable according to literature. For instance, the three-stage diode-clamped converter (DCC) or neural-point-clamped (NPC) converters are in large-power scale category but they are only compatible with PMSGs, and other synchronous machine-based WECSs. Moreover, large-MW-based converters such as active-neutral-point-clamped (ANPC), modular multilevel converter (MMC), etc. are yet at very recent development stages and they are promising to be fully matured by being broadly compatible with wind power industries in the future.

Furthermore, the converter topologies whose operation characteristics and applications are briefly stated in the preceding paragraphs, and which generally include CSC, VSC, DCC, NPC and ANPC are broadly summarized in Table 4 on the basis of their purpose of development, and application and advancement trends that are pertinent to the onshore and offshore wind farm systems.

**Table 4:** Popular PEC designs in modern wind farm applications: Advanced summaries based on literatures.

PEC designs	Operational statuses	Recent trends and advances in PEC designs
CSC [15, 16, 17, 113]	Generally compatible with both onshore and offshore wind farm systems, whereas the most recent developments were largely recommended by studies for offshore applications	CSC-based several various topologies of wind PEC were continuously developed for applications in wind farms of different layouts. On this basis, line communicated CSC topology was proven to demonstrate adequate power conversion capability particularly for onshore wind farm application during the last decades; whereas it is recently incompatible for application in offshore wind power generation. On the other hand, the modified topologies based on various multilevel CSC designs were recently proposed as better candidate to enhance electricity production by improving power reliability and quality. Moreover, PWM-CSCs were most recently reported in literatures as the advanced designs for large-wind power applications particularly in offshore farms.
VSC [10, 20, 114]	Comparatively received the wider acceptances in the onshore wind power applications than the offshore ones, as the onshore-based VSC designs generally operate with optimized costs	Here, the conventional two-level VSCs in back-to-back configurations were developed to meet compatible operation with DFIG-based WECSs of small and medium rated power outputs; and the novel multi-cell two-level VSC topologies were particularly designed to achieve flexible and scalable power rating with PMSG-based WECSs so as to produce electricity at relatively higher-MW levels. However, even though the two-level VSCs based on multi-cell designs were practically proven to show robust wind energy conversion capability, the high material costs associated with the construction of multi-cell converters is still recognized as a main barrier to power production with PMSG-based systems.
MMC [23, 115, 116]	Largely compatible with offshore-based wind farm operating systems, and less common in the applications for onshore energy harvesting due to increasing capacities in its power and voltage levels	This converter technology is generally considered as a recent state-of-the-art in the wind farm industries. Wind farms have been utilizing this technology at varying power scales, and voltage capacities. For instance, MMC topology with power rating of 864 MW, and voltage capacity of ±320kV is presently being implemented at wind farms. Besides, additional topology with more advanced power generating capability was recently unveiled to be under construction for further enhanced application in the future.
DCC [23, 24]	Mostly suitable for offshore wind energy harvesting application, and less applicable for onshore-based wind farms	This converter system was introduced for multi-megawatt scale power generating wind farm application. As it was claimed in one of the recent system modeling-based studies, power generation through the implementation of this converter technology can be significantly enhanced by resulting in increased electricity production and reduced weight of the converter itself.
NPC [26, 117, 118]	More common for offshore wind power generation application than for onshore one	The application of this power converter technology was limited for DFIG-based wind farms though it was proven to show promising candidacy for PMSG-based systems according to literature. Hence, critical consideration is required in this regard to possibly work on design advancement of this converter for its broader application in wind farms.
ANPC [27, 28]	Largely applicable for offshore-based wind farms than for onshore-based ones	Three partial converters that are based on half-bridge modules make up the advanced topology of this converter. Its operation can be severely affected by a short-circuit fault, and some studies proposed methods to deal with this challenge. One of these studies implemented a method based on separation of partial converters so as to limit the impact of short-circuit in a single partial converter. More works are still underway to modify the operation of this converter for enhanced application in the future.

## 3 Output power smoothing methods for variable-speed WECSs

Due to the total nonlinearity of wind speed, the wind energy harvested by WECSs is significantly alternating. A number of considerable issues are yet created by the power ripples including: the grid frequency variation, the real power instability and the voltage flicker at the buses of the power grid. Ultimately, this creates the inferior power quality and disturbances in the WECSs. Furthermore, wind energy has a profound influence on the dynamic performance of WECSs in the course of regular operations and transient faults particularly with the increasing deployment of grid-connected systems. This further complicates the systems failures and, hence, the study of the impact of wind energy on the power grid transient stability has become a highly compelling problem since recently. Accordingly, one of the most important objectives in the application of the WECS technologies is to maintain resilient operation during fault experiences.

In response to the outlined challenges that can severely impact the efficiency and competitiveness of wind power systems, different power smoothing approaches have been introduced in many recent studies in aiming to achieve the various objectives of wind electricity generation by enhancing the performances (power efficiency and cost-effectiveness/competitiveness) of WECSs. That is, based on the diverse power smoothing options, the power smoothing approaches of the WECSs be categorized into two groups: one that can be implemented through the applications of the Energy Storage Systems (ESSs), external hardware devices-based power smoothing systems; and another that based on the computational control algorithms, which can be employed to develop virtual power smoothing systems for regular WECSs that do not rely on the external hardware storage devices (the general comparison between these power smoothing approaches is demonstrated in Table 5 based on various parameters). In this regard, various research studies were conducted to enhance the power performances of WECSs based on the consideration of ESS devices, and with the implementation virtual power smoothing strategies (summaries of the research perspectives based on both approaches are outlined in Table 6). On the other hand, several comparative studies and wind power-related reports unveiled the overall preference of the power smoothing approach without external hardware devices against the ESS devices-based approach on the basis of important criteria. Accordingly, the capital costs of ESS devices-based wind farms are very high compared to the total power production that can be utilized. Moreover, the ESS devices-based WECSs usually lead to the frequent systems' components failures due to the increasing complexities in whole systems’ configurations compared to the WECSs without ESS devices. This can generally cause the systems to fatigue severely and end up in the lower lifespans in addition to the possible reliability degradations. Besides, the application of ESS devices particularly lithium battery technologies in wind farms raise safety concerns as the surrounding environments could be susceptible to the potential explosion hazards from these technologies.

**Table 5:** ESS devices-based WECSs vs. automated systems (without external storage devices)-based WECSs.

Parameters	ESS-based WECSs	Automated system-based WECSs
Overall cost [46, 119, 120]	High	Reduced
Efficiency [45, 121]	High	Moderate to high
Structural load [122, 123, 124]	Complex	Simplified
Reliability [125, 126]	Affected	High
Safety hazards [46, 127]	Probable	improbable
Lifespan [44, 128]	Affected	Improved
Level of development [66, 129]	Relatively matured	Emerging
System's capacity (in MW) [119, 121, 130]	Large	Medium to large

**Table 6:** Summaries of research perspectives with ESS- and non-ESS-based (automated) power smoothing approaches.

Smoothing approaches	Objectives of the studies	Proposed power systems	Employed methods	Ref.
ESS devices	Studying the feasibility of a wind power installation with application of energy storage technology	PMSG-based power plant	Battery energy storage device	[131]
	Aiming at optimizing the investment costs of energy storage device so as to maximize its benefits	Large-scale wind farm	Battery energy storage device	[132]
	Aiming at sizing a large-scale energy storage system based on a parametric analysis in the application to smooth power supply based on high-scale grid integration	Large-scale wind farms	Compressed air energy storage device	[133]
	Investigating the use of energy storage technology for decoupling a power converter from electricity and smoothing its power output	Wind energy converter	Compressed air energy storage device	[134]
	Utilizing a real world data to simulate a power system operating with energy storage device	Wind farm	Flywheel energy storage device	[135]
	Reducing stochastic fluctuations of wind energy	Wind turbine	Flywheel energy storage device	[136]
	Mitigating wind energy fluctuations and augmenting power production	Large-scale MW wind farm	Hybrid energy storage device (compressed air and flywheel)	[137]
Automated control strategies	Enhancing power quality; Increasing power production	PMSG-based WECS	MPPT algorithm; Continuous switching control that applies sliding mode controller	[103]
	Improving power quality and reliability by avoiding the necessity for extra hardware storage devices	MW-level PMSG-based wind power plant	Swap control scheme	[102]
	Maximizing power yield; maintaining the frequency and amplitude of the system's output voltage	PMSG-based WECS	MPPT algorithm combined with pitch control strategy	[138]
	Sustaining the dynamic system frequency so as to maintaining the MPPT operation	DFIG-based wind farm	A two-phase short-term frequency response (STFR) scheme	[139]
	Regulating the power transmission between the wind energy harvesting system and the load by developing a static transfer switch	9 MW wind farm	Pitch angle control based on fuzzy logic	[43]
	Developing the system with strong robustness and adaptability by controlling rotor-side PWM converter – realizing power decoupling control objective	DFIG-based wind power generation	Auto-disturbance rejection control (ADRC)	[140]
	Tracking the MPP by controlling the rotor side VSC – allowing independent control of the generated active and reactive power along with the rotor speed	DFIG-based wind turbine	Novel intelligent control (NIC) scheme	[141]

Consequently, the cost-competitive and high-performing power smoothing approach that does not involve ESS devices, and that which can be implemented without requiring the external hardware devices other than (for example, MPPT algorithms and internal controllers) was recently opted to develop automated systems for various WECSs. Based on the specific methods of virtual power smoothing approach, different algorithms can be designed to regulate various operational parameters of generators and PECs in WECSs so as to achieve the power smoothing objectives. Recently, a dual objectives-control method has been introduced to lessen the torque fluctuations of the turbine shaft that depends on the frequency separation principle. The real current regulation, the generator torque regulation, real and reactive power regulation, and independent pitch regulation strategies have been employed to settle the generator output power disturbances. In the case of PECs, the special features of rotor tied back-to-back voltage source PWM converter control, comprising minimized flicker, variable speed fixed frequency application, self-standing control capabilities for real and reactive powers, and comparatively reduced converter cost and power losses have drawn the researchers' and manufacturers’ attentions across the world. Furthermore, robust and high-performing controllers are needed to be built in WECSs to ensure reliability, enhance efficiency, and eliminate costs. Further, section 4 broadly focusses on the computational algorithms-based output power smoothing approach (automated control strategies) that can be particularly applicable for variable-speed WECSs with DFIG and PMSG operations.

## 4 Automated control for prominent variable-speed WECSs

Control strategies empower WECS to meet the required operation standard by enhancing wind energy harvesting capability, minimizing energy costs, increasing the lifespan of WECS subsystems, simplifying structural loading, decreasing turbine downtimes, and proving an outstanding dynamic and steady-state capabilities. Yet, the most prominent variable speed WECSs such as those based on DFIG, and PMSG are a blend of aerodynamic, mechanical, electromagnetic, and electronic systems, and consequently, management of the numerous subsystems under combination of steady and transient states is challenging. In particular, the growing interest in the grid-connected wind power development has led to further rigorous grid codes, which determine that the WECS must stay linked to the system even under a fault experiences and, hence, it should offer reactive currents to compensate the grid voltages.

**Figure 8:**
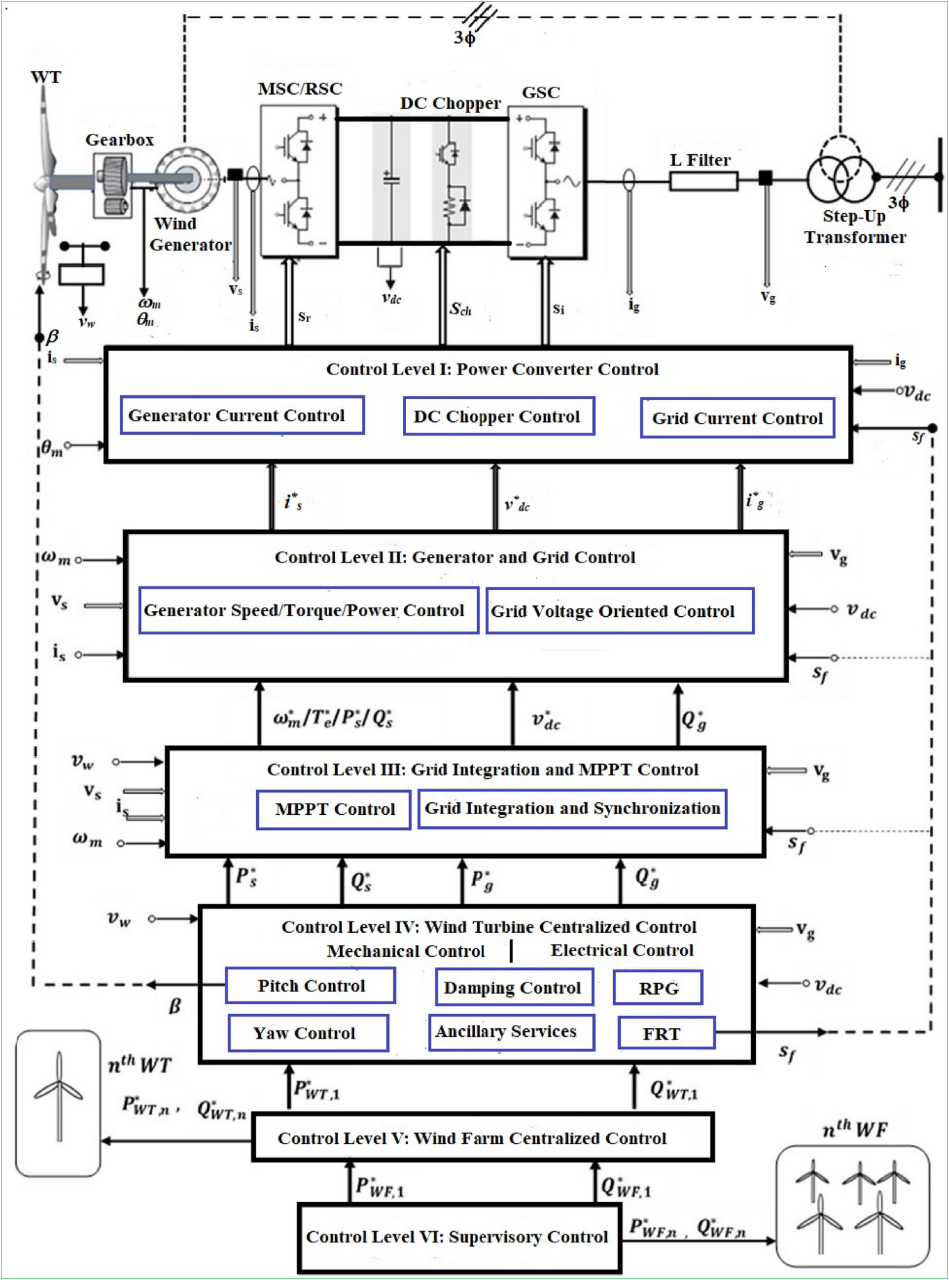
Schematic illustration of all-inclusive control strategy for variable-speed WECS.

The schematic illustration of the all-inclusive power systems regulation strategy for the advanced wind energy harvesting technologies (WECSs) is depicted in Figure 8. The discussion provided under this subtitle is pertinent to variable-speed WECSs that include DFIG, and PMSG. The stator and rotor couplings of DFIG WECS are represented by the dotted lines. The energy harvesting system in DFIG and PMSG is transformed by RSC + GSC and MSC + GSC, in their respective order. The WECSs mostly comprise six control levels, in which the Level I control loop includes rapidly changing parameters and the Level VI control loop consists gradually changing parameters. The stringent regulation of parameters in the Level I loop is crucial to attain the real and reactive power demands enforced by the supervisory control in the Level VI control loop. The control loops additionally inspect standard and anomalous performance of WECSs. Under this subtitle, the control strategies for mechanical and electrical energy harvesting subsystems are analyzed descriptively in the subsequent paragraphs.

As it is depicted in Figure 8, throughout grid irregularities, the FRT control in the Level IV loop accommodates a fault enable signal $s_{f}$. The mechanical and electrical control units in the Level I to IV loops integrate for superior control capability throughout grid irregularities. For instance, at times of grid faults, the GSC holds on to delivering real power and generates reactive power to the grid, the pitch control unit begins operating to slowdown energy harvesting process, and the DC chopper begins responding to halt the DC-bus voltage from surpassing the maximum specification range. The signals response from the WECSs that include grid voltages $v_{g}$, grid currents $i_{g}$, generator voltages $v_{s}$, generator currents $i_{s}$, DC-link voltage $v_{dc}$, generator rotational speed $\omega_{m}$, rotor position angle $\theta_{m}$, and wind speed $v_{w}$ are employed by different control loops. For DFIG WECS, the rotor currents are regulated besides others. The regulation standards are attained by producing ideal gating signals $s_{r}$, $s_{i}$, and $s_{ch}$ for the MSC/RSC, GSC, and DC chopper, sequentially.

The high-level supervisory control (Level VI) dispatches real and reactive power demands to individual wind farm linked to the grid. The first wind farm embraces $P_{WF,1}^{*}$ and $Q_{WF,1}^{*}$, and the nth wind farm embraces $P_{WF,n}^{*}$ and $Q_{WF,n}^{*}$ demands from supervisory control. The power demands from the Level VI control is embraced from the wind farm centralized control (Level V). The wind turbines are linked to the wind farm centralized control by coupling networks to divide the real and reactive power production levels. The wind farm centralized control stands cautiously ready to supervise the wind turbines so that the P and Q references enforced by the top Level VI control loop are satisfied every time. The aerodynamic interaction of the wind turbines is also surpassed by the wind farm centralized control.

**Figure 9:**
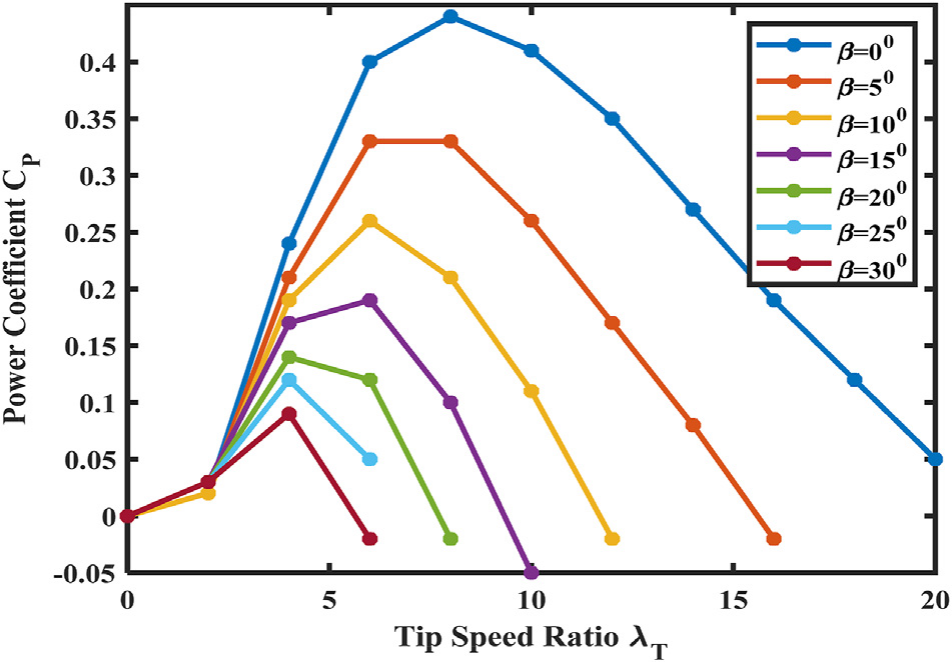
$C_{P}$ versus *λ*_*T*_ curve with different β values.

As illustrated in Figure 9, the wind turbine centralized control (Level IV) comprises systems of mechanical and electrical controls. The pitch control and yaw control are totally incorporated in mechanical control, while RPG and FRT match electrical control. By integrating different mechanical and electrical control units, the wind turbine centralized control supplies real power reference $P_{s}^{*}$ for wind turbine MSC ($P_{s}^{*}$ and reactive power reference $Q_{s}^{*}$ for RSC), together with $P_{g}^{*}$ and $Q_{g}^{*}$ to the GSC. Under regular grid operations, $Q_{g}^{*}$ is adjusted to zero to preserve unity grid PF in PMSG WECS. In DFIG WECS, grid PF is regulated via $Q_{s}^{*}$ command while adjusting $Q_{g}^{*}$ to zero [8].

Current variable-speed WECSs employ a pitch strategy to adjust the spinning of blades in their longitudinal axis. Like it is depicted in Figure 8, when pitch angle β magnifies, $C_{P}$ subsides together with the harvested wind power, and the generator power returns to the actual value.

The Level III control loop embraces maximum energy harvesting, commonly known as MPPT, grid integration, and synchronization. The control system for a GSC performs to synchronize and integrate grid by making use of a phase-locked loop (PLL). The product of the grid interconnection system is the input DC-bus voltage $v_{dc}^{*}$ and input grid reactive power $Q_{g}^{*}$. For a specified grid voltage size $v_{dc}^{*}$ is conventionally set to be fixed as per the desired specification index of a GSC [142].

**Figure 10:**
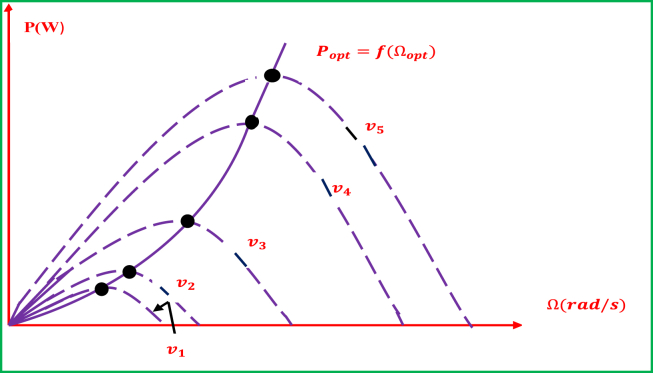
Typical representation of the wind turbine in the plane (power, rotational turbine speed) for modern WECSs.

The typical of the ideal power of a wind turbine is entirely hard to predict and “bell-shaped”. The WECSs should track the possible peak powers for all wind speeds, which is corresponding to tracking the ideal rotational speed. Figure 10 depicts the typical curves of the wind turbine in the plane (power, rotational turbine speed). Each curve correlates to a wind speed $V_{v}$. The peaks of these properties introduce the desired ideal points, which can be represented by a curve called the ideal/optimal power curve and is mathematically defined as [92]:

$$P_{opt}=C_{P}opt\left(\lambda_{opt}\right)\times \frac{\rho \times \pi \times R^{2}\times V^{3}}{2}$$

where: $C_{P}opt\text{:}$ Optimum Power Coefficient; *λ*_*opt*_: Optimum Tip Speed Ratio of turbine blades; ρ: Density of the air; R: Length of turbine blades; V: Wind Speed.

The WECS demands an intelligent tracking of the ideal power curve such that to perform an advanced operation. To achieve this, MPP should be employed. The mechanism of MPPT control involves in regulating the electromagnetic torque so as to transform the mechanical speed in a manner that results in increasing the electrical power production.

**Figure 11:**
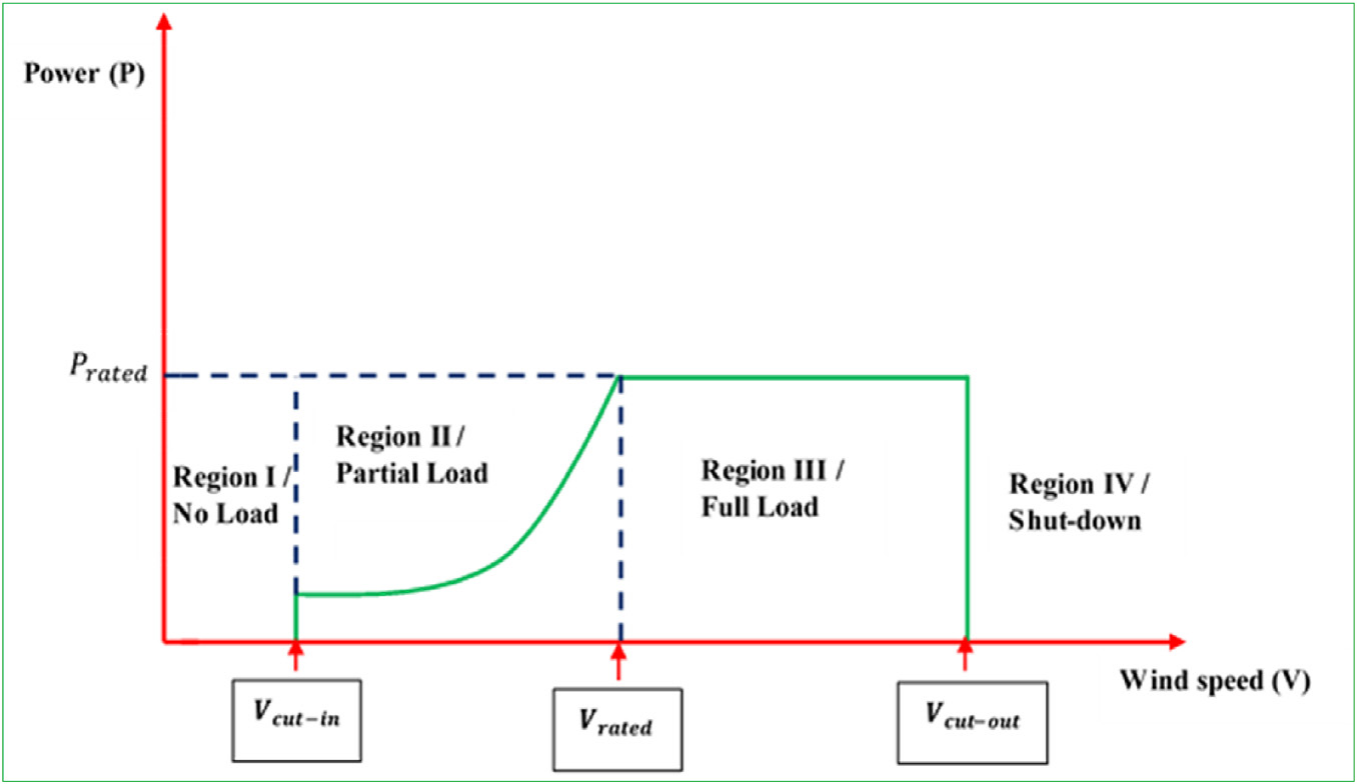
Ideal power versus wind speed characteristics.

There are four performance regions of the variable-speed WECSs and these can be demonstrated by Figure 11. At Region I, wind speeds are too low and inadequate to run the WECSs and generate power whereas at Region II, the wedge angle is remained fixed, and the regulation of the electromagnetic torque will be enacted in a way to harness the high possible energy for individual WECS (by MPPT strategy). In this region, the generator power curve retains a swift progression. At Region III, the generator speed is remained fixed at its peak in contrast to an acceptable torque. The rise in the wind speed results in a reduction in the coefficient $C_{P}$ and a gradual rise in the revived power. When the peak of the power generator is attained, the angle of the blades (pitch) is adjusted (Passage from $\beta_{1}$ to $\beta_{2}$) so as to deteriorate the coefficient $C_{P}$. In Region IV, when the wind speed sharply goes up $V_{M}$, an automatic apparatus is employed to shut the WECS (No electricity generation) so that to avert damage.

More importantly, to realize maximum-energy harvesting and supply the electricity to the grid, the control strategies listed below should be implemented particularly in large scale-power WECSs:

⁃ MPPT for each wind-speed range [143].
⁃ Net DC-bus voltage regulation to meet acceptable operation standards for the GSC [144].
⁃ RPG to match the grid codes [145].

A veracious regulation of wind electric machines and power converters is indispensable to attain the outlined control strategies above. The MPPT strategy is executed by the MSC/RSC, while the GSC monitors the remaining two strategies. Level II control generates the input machine/generator and grid currents ($i_{s}^{*}$ and $i_{g}^{*}$), whereas the Level I control generates reference signals ($s_{r}$ and $s_{i}$), so as to the output machine and grid currents ($i_{s}$ and $i_{g}$) track their inputs ($i_{s}^{*}$ and $i_{g}^{*}$) strictly. In addition, the power transport across the electric machine and utility grid is strictly monitored by Level I control at times of both standard and irregular operations. Under the grid irregular operations, the excess power across the machine and utility grid is transferred to the resistive load via a DC chopper, hence transforming the rotational power of the turbine system into heat. The control unit of the DC chopper interactively determines the portion of power to be transferred to the resistor. The DC chopper control component detects the disturbance signal $s_{f}$ magnitude and supplies the reference signal $s_{ch}$ to the DC chopper so as to the DC-link voltage $v_{dc}$ does not transcend the specified maximum range $v_{dc}^{\max}$.

### 4.1 Power control design strategies for wind farms

Most often, the principles and applications of conventional hard control and standard (hard and soft) control designs have been proposed in multiple research works for: achieving the maximum power extraction from wind, alleviating fatigue loads on WECSs, and maintaining output power dynamic stability according to power quality standards. However, each control method has its own capabilities and limitations (comparisons of different control strategies are given in Table 7). For example, the conventional control design that is based on PID is not intricate and offers reliable operation, but it functions this robustly only for linear models of WECSs. Hence, since WECSs have mostly nonlinear characteristics, conventional control design not be applicable well for a broad range of operations. On the other hand, nonlinear control designs such as standard hard and soft control methods generally perform better than linear or conventional (PID) control method but still both hard and soft control designs have their own specific favorable operational characteristics and drawbacks. Hard control design includes PID (conventional), SMC (standard), adaptive control (standard), etc.; and soft control design consists of FLC, NNC, GA, etc. As it has been already indicated, standard hard and soft control design strategies have proven to be efficacious over a broad spectrums of WECSs operating regions, but there is no well-defined offset between two contradicting control objectives (i.e. maximum power conversion and minimum fatigue damage) when individual strategies are to be implemented. For instance, SMC design strategy is entirely efficient at modeling errors and instabilities in managing the energy conversion operation, but it usually causes to introduce the chattering problem in WECSs, which could eventually deteriorate the lifespans of the systems’ mechanical components and degrade output power qualities. On the other hand, FLC, NNC, GA, etc. design strategies can smooth the complex conditions whose characteristics are unpredictable, inaccurate or have maximum degree of nonlinearity; and yet, these strategies usually demonstrate only moderate efficiencies in managing the power conversion processes.

**Table 7:** Comparison of typical control design strategies for modern variable-speed WECSs.

Control strategies	Control design	Efficiency	Reliability
PID [146, 147, 148]	Simple	Poor	High
SMC [149, 150, 151]	Complex	Excellent	Low
FLC [72, 152, 153]	Simple	Moderate	High
Hybrid (SMC + FLC) [154, 155, 156]	Moderate	Excellent	Moderate
MBPC [20, 59, 157]	Moderate	Excellent	High

**Figure 12:**
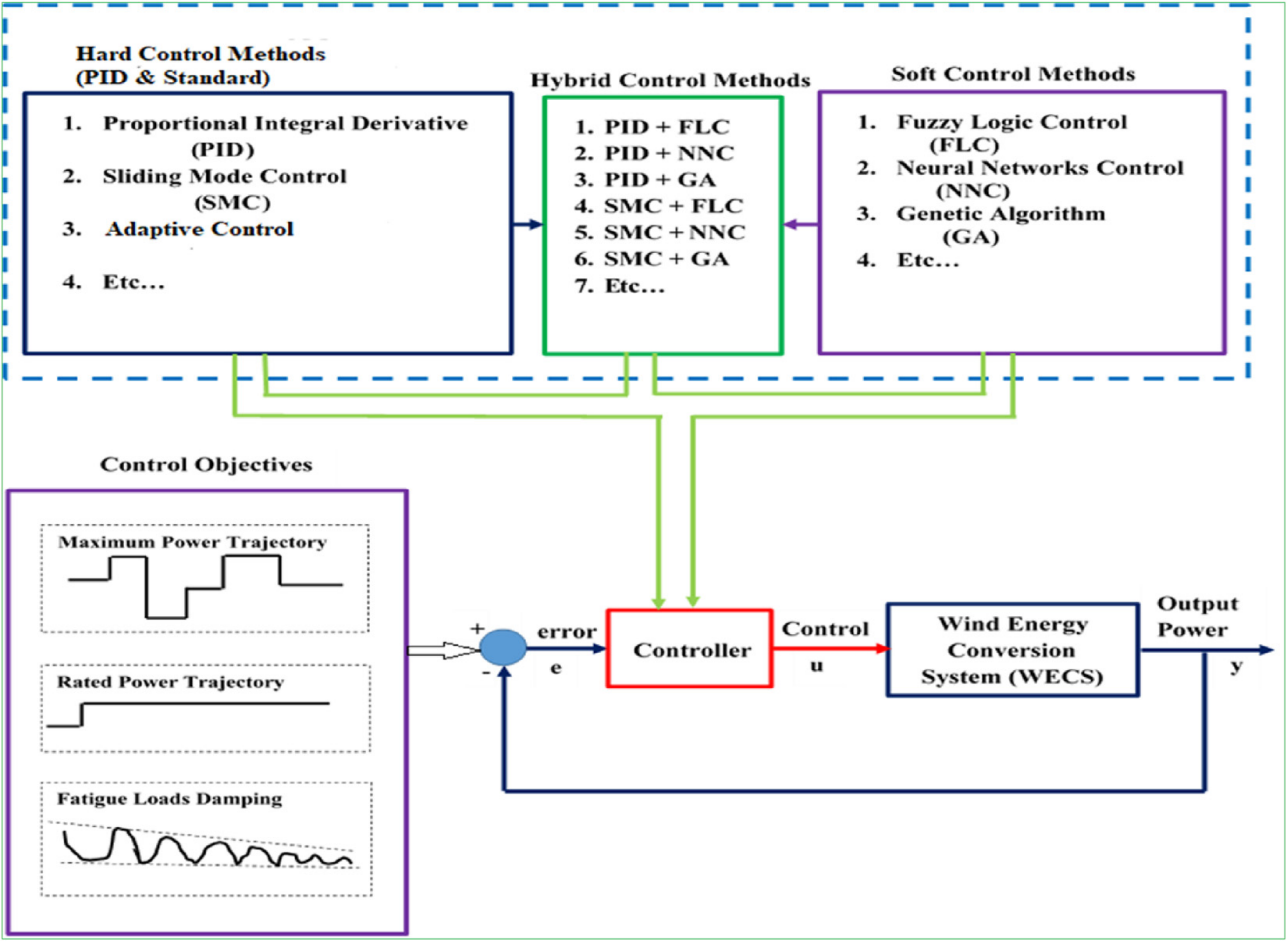
Hybrid control strategies.

**Figure 13:**
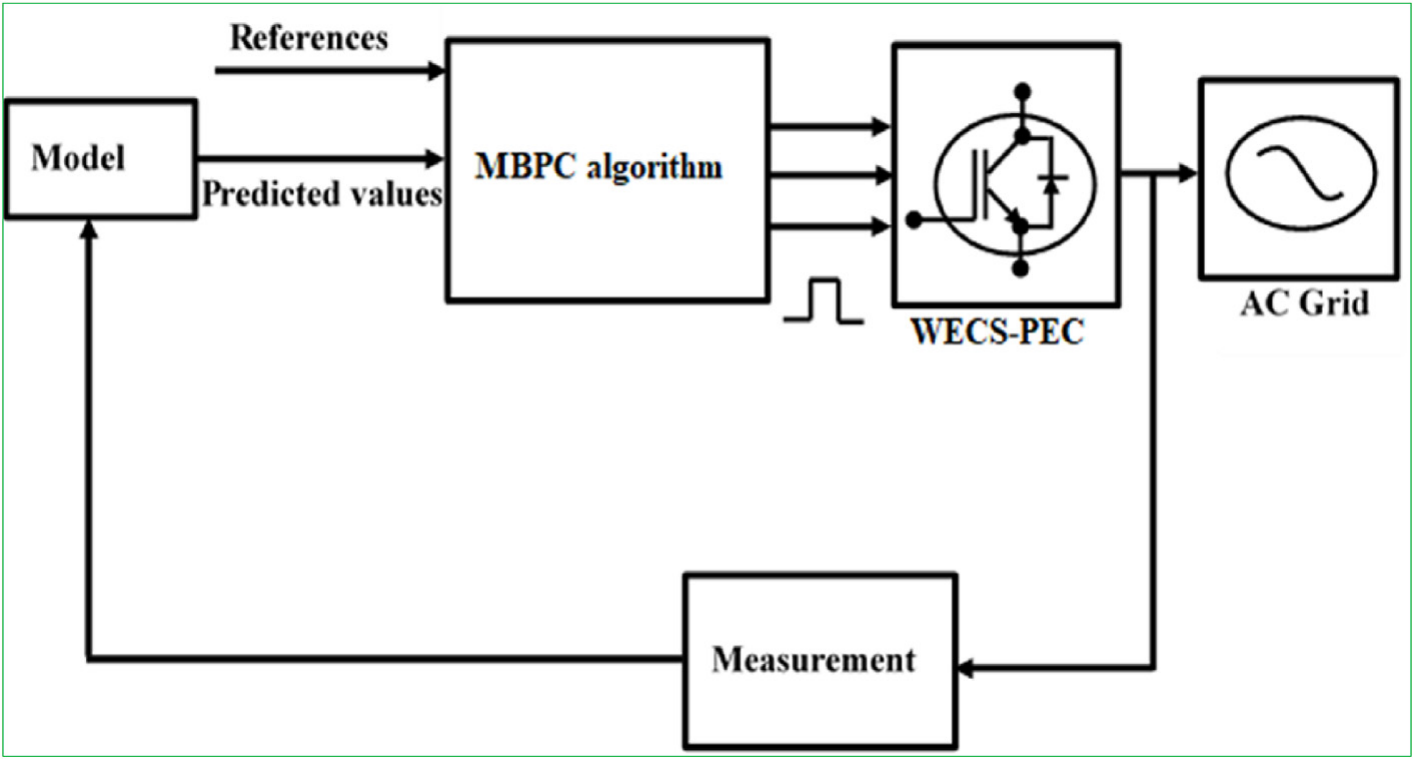
Model-based predictive control strategy.

More interestingly, hybrid or fusion control, and model-based predictive control (MBPC) design strategies are quite appealing due to their unique features that can circumvent the limitations posed by the characteristics of individual strategies including PID, SMC, FLC, etc. Hybrid control design can be developed by the combination of hard and soft computing strategies as illustrated in Figure 12. This control design strategy can improve the dynamic efficiency of the WECSs by minimizing the systems’ complexities along with enhancing output power stability; and yet, this strategy was reported to require additional high-cost to develop control system for the wind farm application [158]. On the other hand, MBPC design strategy was reported to have a number of attractive features that make it more desirable for the advanced control of the PECs-based power generation systems; for instance [20, 56, 159]: it is able to handle well multivariable systems, constraints imposed on the systems are also dealt with satisfactorily, online optimization can be achieved, it is not too intricate to develop control systems, and cost optimizations can be better achieved during operation. Block diagram for MBPC design is illustrated in Figure 13. To achieve the objectives of control design, the MPC strategies execute the following operations [160]: measure the current state of the WECSs to control; predict the trajectories of the WECSs to control from the current state and for a group of specified reference (control input) signals; select reference (control input) signals that reduce the MBPC algorithm, which potentially relies on the predicted trajectories and the references (control inputs); and apply the PEC signals for the finite amount of time.

Moreover, recent studies largely proposed MBPC design strategy along with various power systems control inputs, and optimization models in aiming to increase power production of different wind farms, and WECSs/individual turbine technologies across the world. As it can be seen from the summaries presented in Table 8, these studies claimed varying levels of increments in the power production by the implementation of MBPC strategy with different models of wind farms and turbine technologies, and under consideration of various control inputs. These increments generally seem to depend on the models of the proposed wind farms or turbine technologies, and the considered control inputs for optimizations. For instance, a wind farm model with unspecified number of turbines was studied to maximize its power production based on the optimization of blade pitch angle and tip speed ratio by researcher in [61], and power maximization of 0.4–1.4% was reported. Similarly, DFIG, and PMSG turbine technologies-based power maximization were independently studied in [161] and [60] with different layout models, where power production of 2% and 20% were claimed to be maximized respectively. Furthermore, axial induction factor [162], and yaw angle –and– blade pitch angle [163] were considered to be optimized as the control inputs in aiming to maximize the power production of two wind farms with different layout models, and varying scales and ranges of increments indicating 2–8% and 30.4–33.2% were respectively reported to be achieved. The analyses conducted so far can also apply to the rests of the studies presented in Table 8. Based on these study results, it can be generalized that application of MBPC strategy for the real-world wind farm optimization lead to maximization of power production.

**Table 8:** Summaries of recent research reports on wind power maximization by using MBPC strategy.

Control strategy	Proposed power system model	Control inputs	Power maximization	Ref.
Model-based predictive control	Wind farm with unspecified number of turbines	Blade pitch angle and Tip speed ratio	0.4–1.4%	[61]
	DFIG-based offshore wind farm	Rotor angular speed	2%	[161]
	PMSG-based turbine	Rotor angular speed and Axial induction factor	20%	[60]
	A 2×3 wind farm layout	Axial induction factor	2–8%	[162]
	Wind farm composed of 100 turbines	Yaw angle and Blade pitch angle	30.4–33.2%	[163]
	Two wind farms composed of 2 turbines; and 9 turbines	Tip speed ratio and Blade pitch angle	Up to 38%	[164]
	Wind farm layout composed of 12×6 turbines	Thrust coefficient	8–21%	[165]
	Wind farm layout composed of 4×4 turbines	Thrust coefficient and Yaw angle rate	Nearly 30%	[166]
	Wind farm composed of 9 turbines	Yaw angle	7–11%	[167]

## 5 WECS design development approaches

For the technologies as sophisticated as the WECSs, the capability to mimic the real-world systems (mechanical, electrical, hydraulic, etc.) and control systems under a unified framework is indispensable to the design development process. In line with this consideration, MBD has been recently introduced by researchers as the effective and efficient approach for modeling structurally sophisticated energy conversion technologies particularly that of wind [168]. It allows engineers to incorporate specifications into the design development process, to develop design at the system level, and to predict and enhance overall system performance with no need to necessarily relying on hardware/physical prototypes [169]. In addition, it accelerates design development process, enhance systems, and enables to minimize design development costs. For instance, the wind turbine technology developers that employ MBD methodology achieve substantial savings when compared to traditional methods as reported by recent studies. MBD has further several advantages against conventional design (the comparison between conventional design and MBD methods is detailed in Table 9.) The major savings can be attained from healthier requirements analysis combined with early and continuous testing and verification. As requirements and designs are developed applying models, defections are recognized particularly at advantageous time, while they are at stages of development costing less to handle. Furthermore, MBD relies on the overall simulation model of the machine and the associated control algorithm under development, i.e. the control algorithm is developed in a simulation platform that offers early validation capabilities by simulation experiments. WECS models have a distinguished role in MBD, and they are mainly designed in MATLAB/Simulink, which is widely a desirable platform particularly for simulation WECSs’ control designs [170].

**Table 9:** Conventional design development Vs. Model-based design development.

Conventional Development	Model-Based Design (MBD)
Requirement Documents [171]: ⁃ Hard to analyze ⁃ Arduous to control as they change Physical Prototypes [172]: ⁃ Deficient and costly ⁃ Do not allow rapid iteration ⁃ System-level testing is ineffective Manual Coding [173]: ⁃ Inefficient ⁃ Causes errors and discrepancy ⁃ Hard to reapply Traditional Testing [174]: ⁃ Design and integration problems detected late ⁃ Ambiguous to feed insights back into the design process ⁃ Ascribable Experimental Arrangement [175]: ⁃ Conspicuous ⁃ Makes good understanding of physical reality	Executable Specification [176]: ⁃ Not difficult – simple to comprehend ⁃ Systems optimization– modeling overall system environment ⁃ Sharing of models to enhance learning and collaboration ⁃ Safe validation and test configuration Automatic Code Generation [177]: ⁃ Avoid deficiencies from manual-coding ⁃ Regenerate smoothly for various objectives Continuous Test and Verification [64]: ⁃ Sense defects fully in design process ⁃ Minimize relying on physical prototypes ⁃ Reapply tests throughout the design buildout process Platform independence [63]: ⁃ be code implementation time has to be optimized ⁃ Device-specific modulations need to be included Real-time Simulation [169, 178]: ⁃ Not fully represent real physical systems

### 5.1 Key elements and methodology of MBD

**Figure 14:**
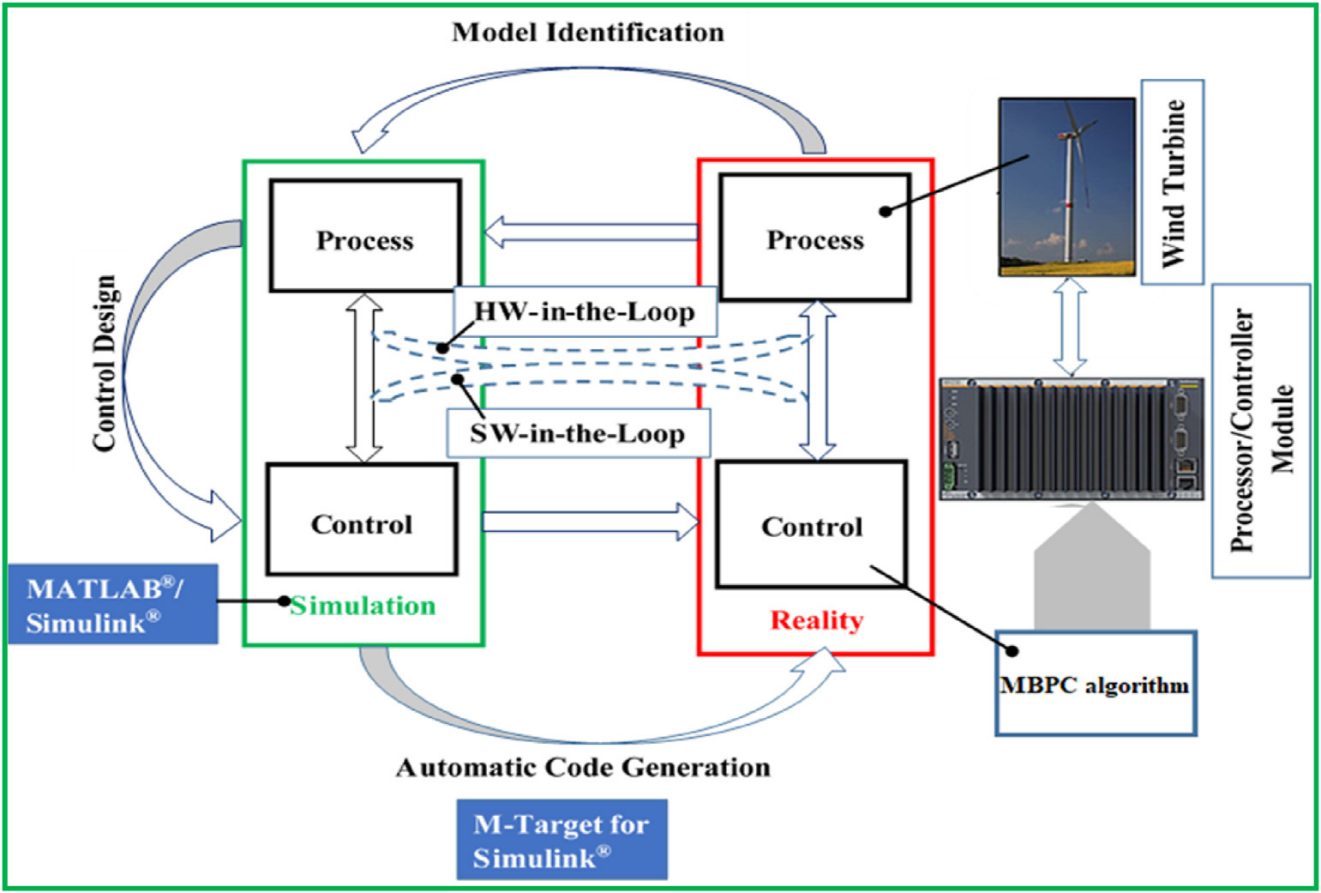
Rapid Control Prototyping (RCP) development process of WECS.

The improvement of WECSs is mainly determined by their efficacious operation, which can be profoundly enhanced by self-regulating approach, which is model-based control. Study on this approach has been carried out in recent years and it is now highly introduced in in the wind energy industry as a way to meet challenges in energy harvesting and power networks. The various stages of MBD are illustrated in Figure 14. In this regard, RCP [179] becomes largely an important technology in the MBD workflow for feeding control algorithms into a real physical systems. Furthermore, RCP comprises a devoted high-performing real-time target computer and the associated software environments with which the control algorithms could be validated effectively in the real physical system. The MBD/RCP development process begins with modeling a WECS completely in the software environment, namely MATLAB and Simulink. Different block models mimic the real-world WECS comprises of mechanical, electrical, and hydraulic components. These WECS components are accompanied by models of aerodynamic loads and are simulated by various input parameters that include wind speed, wind direction, etc.

In the rapid prototyping process, the WECS simulation model produces C code. This means that the C code is produced from the control algorithms or software-in-the-loop (SIL) that is developed in the model for the supervisory control unit, and this generated c code can be employed for two objectives. First, it can be fed into high-performance controller device system. To test this control code and the controller device, hardware-in-the-loop (HIL) tests be implemented in place of the whole hardware frameworks of WECSs. HIL requires employing the WECS models of the hardware systems (mechanical, electrical, and hydraulic) to produce C code and feed it to a real-time target computer. Second, the HIL real-time target computer links to the hardware controller and mimics the characteristics of the real-world WECSs. Consequently, developers can test the control unit over a broader scale of operations than would be effective with the whole hardware frameworks. Eventually, by applying the similar WECS models of the real-world systems as applied during the initial processes of design development, the engineers can also verify and confirm that the produced code operates accurately like it run through desktop simulation.

## 6 Conclusion and future prospects

In this paper, the topologies and features of various WECSs along with their power output smoothing methods, control strategies, and design approaches have been sequentially considered, and the important conclusion can be drawn here. Modern WECSs generally operate as the variable-speed technologies so as to maximize wind power generation by ensuring the production of electricity below the rated power along with minimized loads on the drivetrains. Moreover, in the last few decades, relentless efforts were made by researchers and manufacturers in introducing various enhanced WECS technologies that have already contributed to the globally maximized power production, improved power reliability and quality along with reduced costs of wind energy. In this regard, the DFIG-based wind energy conversion technology is the dominant system largely in onshore wind energy industries, and its high power production per cost performance makes it exceedingly desirable; whereas PMSG-based system has recently become to challenge DFIG system's future global power generation share due its increasingly emerging electrical components that can better smooth the production of electricity with outstanding power capacity, particularly in the case of offshore applications. On the other hand, extensive research studies are undergoing in enhancing the complex structural design, and optimizing the high commercial cost of EESG-based wind energy harvesting technology. Yet, the recent trends in wind power-related engineering studies, and technology deployments indicate the prevalence of DFIG- and PMSG-based systems.

As the main electrical components of WECSs, PECs occupy considerable space in impacting the overall performance of wind power production. Yet, the wind energy conversion performance of PECs depends on their different types of topologies and configurations. For the last several decades, 2LVSC converter topologies in BTB configurations were dominantly opted for applications in wind farm industries due to the reason that they are well proven, efficient and reliable in addition to their compatibility with both DFIG- and PMSG-based WECSs. To be more specific, the conventional 2LVSC (with reduced voltage capacity) is employed for DFIG-based system; and multi-cell 2LVSC (with extended voltage capacity) in BTB configuration is compatible with operation of PMSG-based system. Here, the general limitation of DFIG-based system is caused by the incompatibility of its design structure with the extended converter capacity, and thus it needs to be possibly advanced for applications in the wind farms of increasing power capacities. On the other hand, huge consideration should be intended to optimize the commercial cost of PMSG-based WECS that is particularly associated with its power converter design.

For the multi-megawatt scale wind power generation, PECs with high-voltage capacities (including MMC, DCC, NPC, ANPC, etc.) were being introduced as the viable solutions for wind power industries that primarily employ PMSG systems. Some of these converter technologies were reported to be still at their early stages of developments, and the requirements for further improvements in terms of their sizes, weights, power efficiency, and material/design costs have been indicated to be met in the future. Thus, despite the fact that important milestones were achieved in the WECS technologies in improving the wind-energy extraction efficiencies so far, more significant advances are still required to be made to meet highly optimized electricity generation in the future, which would match with UNCCC's goals (large increase in power production, and significant reduction in costs by 2050 in transitioning from recent trends). To be clear, these [UNCCC] goals were set primarily based on the anticipation that rapid and sequential improvements would take place in enabling technologies. Furthermore, the current trends of wind power generation indicate that more advanced and rapid progresses are required to be made in wind energy conversion-related engineering methods and technologies to smooth transition towards the goals. For instance, immediate consideration should be towards the full developments (design, power efficiency, and cost optimization) of PECs that were recently recognized by studies as the current state-of-the-art solution (MMC), and promising technologies (DCC, NPC, ANPC, etc.) for future applications.

In addition, it can be generally understood that power management system is a core component of a WECS operation as it could be implemented to ensure enhanced (more reliable, efficient, and safe) wind power production along with decreasing electricity costs. As it was indicated through this work, two different approaches be independently employed to achieve some objectives of electricity management: one approach could be built as the external hardware systems (commonly referred to as ESSs) to WECSs, and another could be designed as virtual systems by being embedded in WECSs configurations. The ESSs are usually characterized to have reliably high capacities of storing wind energy that could be used during power peak times; whereas the virtual systems could be internally implemented to further enhance the generators and PECs so that their operations are smoothed stringently, and power demands are automated. Here, the main recent challenge in commissioning the wind farms with application of ESS devices-based WECSs is the high associated capital and operating costs, which need to be significantly optimized in the future through the possible implementation of enhanced strategies. On the other hand, promising study progresses are currently being made to build multi-objectives and automated – output power smoothing system for the regular (non-ESSs-based WECSs) by the implementation of various control algorithms (hybrid, model predictive, etc.) through the MBD methodology. This approach was widely reported in the recent studies to be highly cost-effective during both the development and application of systems compared to ESS devices. Moreover, multiple research results generally indicated that the wind power production can be maximized by the implementation of automated control strategies. However, the broad practical applicability of these strategies in the real-word wind farms is yet to be fully adopted though the most recent advanced studies have been indicating very promising future for full-scale commercial applications.

## Declarations

### Author contribution statement

All authors listed have significantly contributed to the development and the writing of this article.

### Funding statement

This work was supported by 10.13039/501100005872Bahir Dar University Institute of Technology, Energy Center; and Wolaita Sodo University.

### Data availability statement

No data was used for the research described in the article.

### Declaration of interests statement

The authors declare no conflict of interest.

### Additional information

No additional information is available for this paper.
